# Covalent Docking to the Active Sites of Thiamine Diphosphate-Dependent Enzymes

**DOI:** 10.3390/molecules30224427

**Published:** 2025-11-16

**Authors:** Artem V. Artiukhov, Vasily A. Aleshin

**Affiliations:** 1 Department of Biokinetics, A. N. Belozersky Institute of Physicochemical Biology, Lomonosov Moscow State University, 119234 Moscow, Russia; 2 Department of Biochemistry, Sechenov University, 105043 Moscow, Russia

**Keywords:** covalent docking, ligand-coenzyme interactions, thiamine diphosphate-dependent enzymes, gnina, thiamine pyrophosphate, pyruvate dehydrogenase, 2-oxoglutarate dehydrogenase, branched-chain 2-oxo acid dehydrogenase, esterified inhibitors, phosphonate inhibitors

## Abstract

The search for novel low-molecular regulators using molecular docking continues to be crucial for addressing challenges in modern biomedical science. However, the current literature lacks examples of modeling covalent interactions between the ligands being docked and those already present within the proteins, such as enzyme cofactors. This study aims to improve the existing algorithms for modeling such interactions, exemplified by those in thiamine diphosphate (ThDP)-dependent enzymes. Structures containing adducts of ThDP with enzyme substrates or inhibitors are used as protein templates; the putative ligand models are prepared as (R)- or (S)-hydroxy derivatives. The Gnina framework with AD4 or Vinardo favors ligand conformations resembling those found in the protein templates and consistent with their relative inhibitory potentials in experiments in vitro. For example, local hydrophobic regions within pyruvate and branched-chain 2-oxo acid dehydrogenase structures favor the binding of esterified substrate analogs compared to their de-esterified counterparts. The preferred binding of esterified vs. de-esterified ligands is absent or even reversed for 2-oxoglutarate dehydrogenase. As a result, covalent docking of 2-oxo acid analogs to enzyme structures containing ThDP coenzyme offers a predictive capability for protein–ligand complex formation and should be used when inhibitors mimic transition states in enzymatic reactions, as observed with ThDP-dependent catalysis.

## 1. Introduction

Thiamine diphosphate (ThDP) is a ubiquitous coenzyme essential for catalysis in a number of enzymatic reactions in all organisms [1,2,3]. The enzymes using ThDP as a cofactor are often localized at the intersection of multiple metabolic pathways. For example, pyruvate dehydrogenase (PDH), known to catalyze the oxidative decarboxylation of pyruvate to acetyl-CoA, links the glycolysis and catabolism of amino acids that degrade through pyruvate (such as alanine, serine and cysteine) with the TCA cycle and the synthesis of fatty acids and other lipids [2,4,5]. Operating under a variety of regulatory factors and post-translational modifications, PDH thus ensures a metabolic switch between aerobic and anaerobic metabolism. Some sources also indicate that PDH is able to catalyze a similar reaction with the pyruvate homolog 2-oxobutyrate, transforming it into propionyl-CoA [6]. The latter is also an intermediate in the amino acid degradation pathways (such as catabolism of threonine and methionine), reflecting another important role of ThDP-dependent catalysis.

Another ThDP-dependent enzyme, 2-oxoglutarate dehydrogenase (OGDH, also known as α-ketoglutarate dehydrogenase), catalyzes the oxidative decarboxylation of 2-oxoglutarate to succinyl-CoA, which is a rate-limiting step in the TCA cycle [7]. Through 2-oxoglutarate, OGDH participates in the degradation of several amino acids such as glutamate, glutamine, histidine, arginine, and proline. Its importance for the energy metabolism is exemplified by a complex network of allosteric regulators such as calcium ions, NAD/NADH, and ADP/ATP ratios [4,7,8,9,10]. In humans, there are also two OGDH paralogs encoded by the *OGDHL* and *DHTKD1* genes. The latter paralog acts mostly on 2-oxoadipate, which contains an additional methylene group, compared to 2-oxoglutarate. This intermediate of lysine and tryptophan degradation is transformed into glutaryl-CoA [10,11,12,13,14], which is a potential donor of glutaryl moiety in protein glutarylation [15,16], thus gaining more attention in the epigenetics field. Both PDH and OGDH can also participate in protein acylation, producing acetyl-CoA and succinyl-CoA as donors of acetyl and succinyl moieties, respectively [16].

One more mammalian ThDP-dependent enzyme is branched-chain 2-oxo acid dehydrogenase (BCDH), which is responsible for the degradation of branched-chain 2-oxo acids. These are the products of the transamination of valine, leucine, and isoleucine. Ono et al. [17] also demonstrated that BCDH can catalyze the slow oxidative decarboxylation of 2-oxoglutarate and, comparatively faster, the methionine transamination product 4-methylthio-2-oxobutyrate [18]. Similarly to PDH, BCDH also transforms 2-oxobutyrate into propionyl-CoA [6], thus linking the metabolism of branched-chain and other amino acids with energy production [4].

While the ThDP-dependent enzymes mentioned above function within the large multienzyme complexes, there are also mammalian ThDP-dependent enzymes with much simpler oligomeric structures. Of those, transketolase (TKT) is the most important and studied one. The enzyme catalyzes a rate-limiting step in the non-oxidative branch of the pentose phosphate pathway, facilitating the transfer of two-carbon oxo-units between sugar phosphate molecules. Thus, TKT maintains various biosynthetic processes and cellular redox status via NADPH generation [2,19]. Finally, 2-hydroxyacyl-CoA lyase (HACL) catalyzes the elimination of the one-carbon unit (formyl-CoA) from long-chain acyl-CoAs in the fatty acid alpha-oxidation pathway [20,21,22].

**Figure 1:**
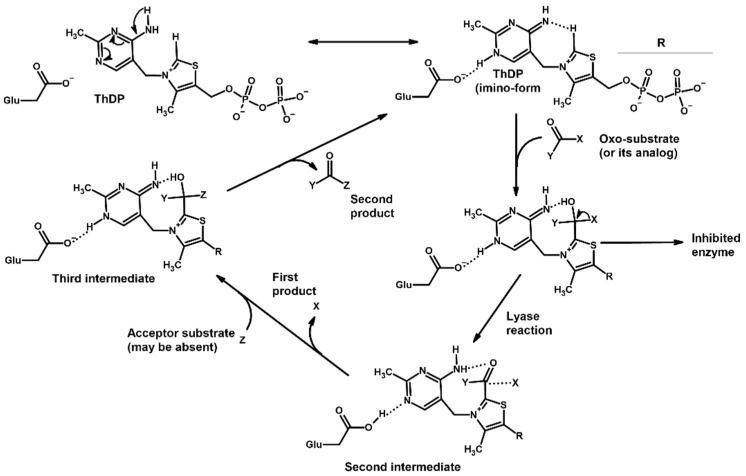
Scheme describing ThDP’s role in the reactions catalyzed by ThDP-dependent enzymes. In case of mammalian PDH: X = carboxyl group, Y = methyl or ethyl group, Z = lipoamide; OGDH: X = carboxyl group, Y = carboxyethyl or carboxypropyl group, Z = lipoamide; BCDH: X = carboxyl group, Y = branched-chain alkyl group, Z = lipoamide; TKT: X = hydroxymethyl group, Y = phosphoglyceryl group, Z = glyceraldehyde phosphate; HACL: X = long-chain hydroxyalkyl group, Y = coenzyme CoA, Z is absent.

These five enzymes, with their various isoforms, constitute the ThDP-dependent enzyme family in vertebrates; however, plants, fungi and various microorganisms possess even more ThDP-dependent enzymes with broad substrate specificity [1,2,23,24,25]. Nevertheless, they all share a similar catalytic mechanism, in which ThDP initially forms covalent interactions with the oxo groups of various substrates, transforming them into their hydroxy group-intermediate states followed by the splitting of carbon–carbon bonds and the transfer of the remaining molecule to various acceptors (Figure 1).

Since all of the mentioned ThDP-dependent enzymes are known to have key roles in central or organism-specific metabolic pathways, they are attractive targets for drug discovery. However, despite the significant advancements in fast computational methodologies—including docking simulations of covalent binding to protein residues, whole protein docking, the development of new software and novel scoring functions, and finally re-estimation of docked positions with convolutional neural networks—to our knowledge, no publicly available protocol currently describes modeling the covalent binding of some ligands to another ligand such as ThDP directly. This binding is nevertheless important for the inhibitors of ThDP-dependent enzymes [5,12,24,26], thus such an algorithm needs to be introduced.

Molecular docking has been a valuable computational tool for visualization of protein–ligand binding as well as the screening and design of novel ligands. In 2025, dozens of software models are available for performing docking experiments, the most popular of which are AutoDock, with its multiple forks, including Vina, Smina, Gnina, as well as GDOCK, Gold, and Glide [27,28,29]. However, almost all of the existing software is designed to model non-covalent binding of ligands, which corresponds to the fact that the existing drugs mostly act as competitive enzyme inhibitors or receptor agonists/antagonists [30,31]. However, recent drug design studies have found that as much as one third of FDA-approved drugs may act via covalent binding to the desired or undesired targets [32,33]. Consequently, more docking software has emerged, simulating covalent bonds, and some of the existing ones, like AutoDock and its forks, have added this functionality as well. This has facilitated the development of new prospective drugs, like irreversible inhibitors of beta-lactamase and various protein kinases [34].

Current techniques for the simulation of ligand binding to the active sites of ThDP-dependent enzymes specifically either involve just the simulation of non-covalent binding near ThDP [35,36], manual introduction of the optimized ligand moiety in the existing template with ThDP but without the actual assessment of potential conformations [26], or docking of the ThDP-ligand adduct as a single molecule replacing the existing ThDP [37,38]. However, the known mechanism of ThDP-dependent catalysis, which requires the formation of a covalent bond between the C2 atom of ThDP and the carbonyl atom of a ligand, necessitates the precise modeling of non-covalent protein–ligand and coenzyme–ligand interactions in close proximity to ThDP itself. The current study fulfills the lack of such a procedure, using the Gnina framework (https://github.com/gnina/gnina/tree/v1.3, accessed on 11 December 2024) with a covalent setup and non-default scoring functions. The described algorithm shows a good performance of covalent docking in terms of RMSD compared to existing protein–coenzyme–ligand complexes. But what is even more relevant when comparing several ligands is that the estimated binding efficiency is fairly proportional to the results of in vitro kinetic studies described in the literature.

## 2. Results

### 2.1. Using Pyruvate Dehydrogenase in a Complex with Thiamine Diphosphate and Acetyl Phosphinate to Validate the Docking Setup

Among the known eukaryotic PDH structures available in PDB, only one contains a covalent ThDP adduct with a substrate or inhibitor: 6CFO [39]. The structure envelops the PDH heterotetramer into a complex with Mg^2+^ ions and the covalent adduct of ThDP and acetyl phosphinate (AcPH), a synthetic analog of PDH substrate (pyruvate) in which the carboxyl group is replaced by the phosphinate one. While the carboxyl group is cleaved during the enzymatic reaction (X in Figure 1), its replacement with the phosphinate group prevents such cleavage, resulting in PDH inhibition. In order to model the binding of the known substrates (pyruvate, 2-oxobutyrate, glyoxylate) and their analogs (AcPH, acetyl (methyl) phosphinate (AcMePH), acetyl phosphonate (AcP) and methyl, dimethyl, and ethyl phosphonoderivatives of the latter (AcPMe, AcPMe_2_, and AcPEt, respectively)) to PDH, their models were prepared as hydroxy-derivatives (Table 1), which should be present during the formation of the transition state or its mimics (Figure 1).

Since the formation of these hydroxy-derivatives during the reaction results in two potential stereoisomers, structures of both (R)- and (S)-hydroxy-derivatives were initially prepared. To dock the ligands to ThDP within PDH, several common software models which have covalent docking setups were tested, namely LeadFinder [40], AutoDockFR [41], GDOCK/HADDOCK (https://github.com/gdocking/gdock, accessed on 29 April 2025, [42]), Smina [43], and Gnina [44,45]. Among all those, only LeadFinder and Gnina were able to actually simulate covalent bonding between a specified ligand atom and a specified coenzyme atom, while the rest struggled with parametrization of atoms that did not correspond to standard amino acids list. However, unlike LeadFinder v.1.2.40 (developed by BioMolTech, Inc., Toronto, ON, Canada), Gnina software is open-source, can implement multiple parameters, and is not as outdated. Moreover, despite only working in a Linux environment, Gnina can be installed in the Windows Subsytem for Linux (WSL) shell, where it is easily understandable. Thus, further work was continued using Gnina in WSL.

**Figure 2:**
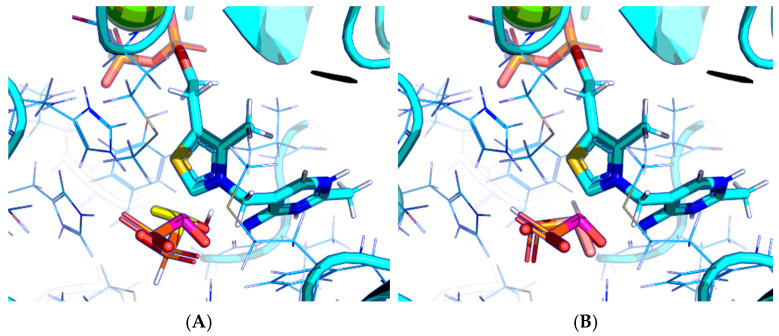
Visualization of covalent docking of AcPH to ThDP within the PDH structure using Gnina with default scoring function. Only conformations with the lowest binding energy are shown. Carbon atoms of the PDH protein template from PDB ID: 6CFO (including ThDP and nearby amino acid residues) are colored in cyan; carbon atoms of AcPH initially present in the PDH structure are in magenta; carbon atoms of docked AcPH as (R)-hydroxyderivative (**A**) or S-hydroxyderivative (**B**) are in yellow and salmon, respectively; non-carbon atoms are colored according to a standard color scheme. Amino acid residues within 6Å of the docked ligands are shown as wires.

The results demonstrate that, first, Gnina predicts much more favorable binding (−2.02 kcal/mol for top conformation) of the R-form of the ligand (hydroxy-derivative of AcPH) vs. its S-form (−0.59 kcal/mol). This result is in good accordance with the template structure (RMSD = 1.27 Å in Figure 2A vs. 1.50 Å in Figure 2B). In addition, most of the ThDP enzymes have a chirality in their side reactions, carboligation [46], predominantly forming S-hydroxyketones or S-hydroxy acids. Their conformations correspond to those of R-AcPH due to the different priority of the carboxyl and phosphinate groups in IUPAC nomenclature. PDH also follows this rule, forming 10-times more S-acetoin than its R-enantiomer [46]. Thus, when using similar covalent docking algorithms for ThDP-dependent enzymes, one should either work with structures of both chiral forms of hydroxy-derivatives of ligands, or check which specific chiral form may be favorable for mimicking the intermediary state of the enzyme-catalyzed reactions.

**Figure 3:**
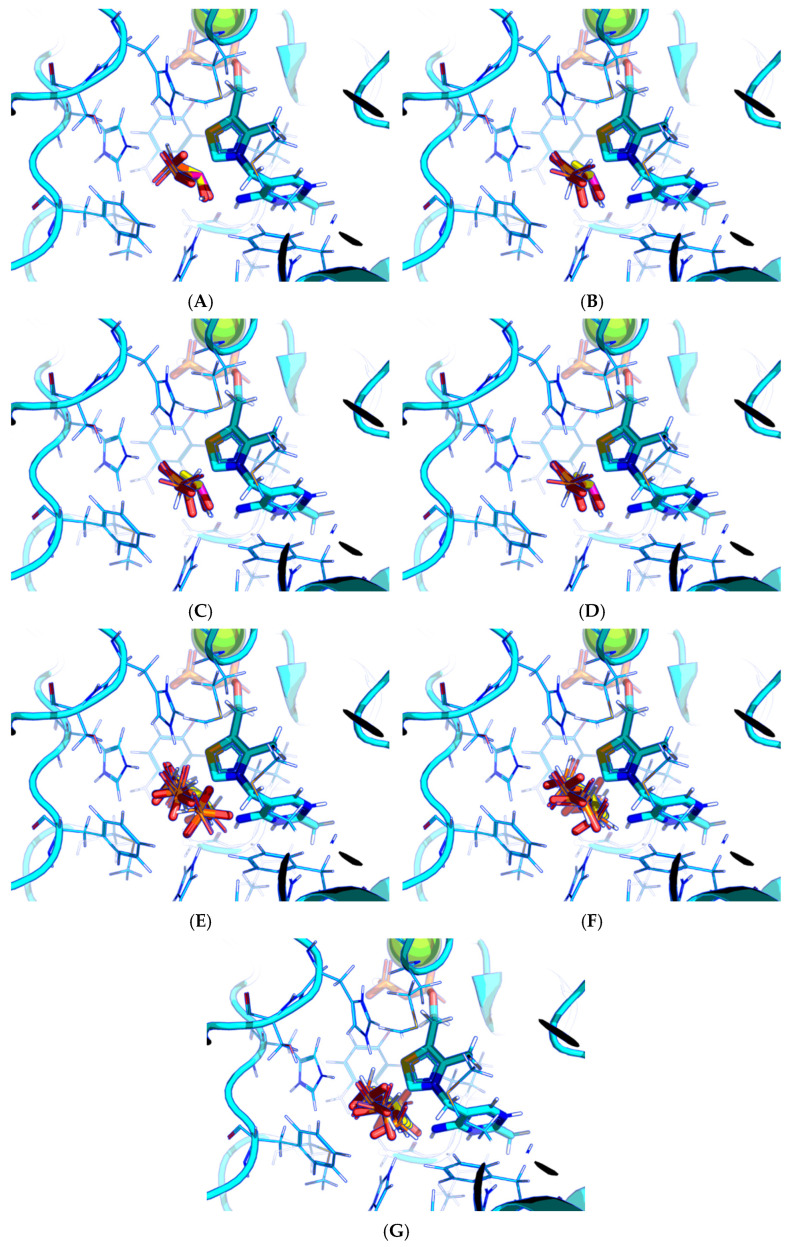
Comparison of favorable docking positions of AcPH to ThDP-PDH complex using Gnina with different scoring functions. (**A**) Default function; (**B**) Vina function; (**C**) Vinardo function; (**D**) AD4 function; (**E**) Dkoes function; (**F**) Dkoes_fast function; (**G**) Dkoes_old function. Only conformations with negative calculated binding energies are shown. Atom color scheme is the same as in Figure 2A. Amino acids residues within 6Å of the docked ligands are shown as wires.

Next, the docking protocol was modified in order to position the carbonyl atom of AcPH in a specific position relative to the C2 atom of ThDP based on the coordinates of the AcPH template initially present in the PDB ID:6CFO structure. This resulted in the docked ligand conformations being quite similar to the templated ones. In addition, all scoring functions supported by Gnina v 1.3 were tested. Among them, Vina and Vinardo give the same result as the default one (Figure 3A–C), however AD4, with more explicit electrostatic interactions and desolvation parameters [47,48], seems more prominent, favoring AcPH conformations with the smallest possible deviation from the template (Figure 3D). Based on the known improved predictive capabilities of Vinardo scoring vs. Vina tested in Smina fork [49], we have decided to show the results from both AD4 and Vinardo scoring for further evaluation of Gnina. Other available functions resulted in a lot of ligand conformations with negative binding energies (Figure 3E–G), with the top conformations usually placing the methyl group of AcPH where the hydroxyl group interacting with the 4′-amino group of ThDP should be.

### 2.2. Comparison of the Binding Efficiency of the PDHC Ligands In Silico and In Vitro

**Figure 4:**
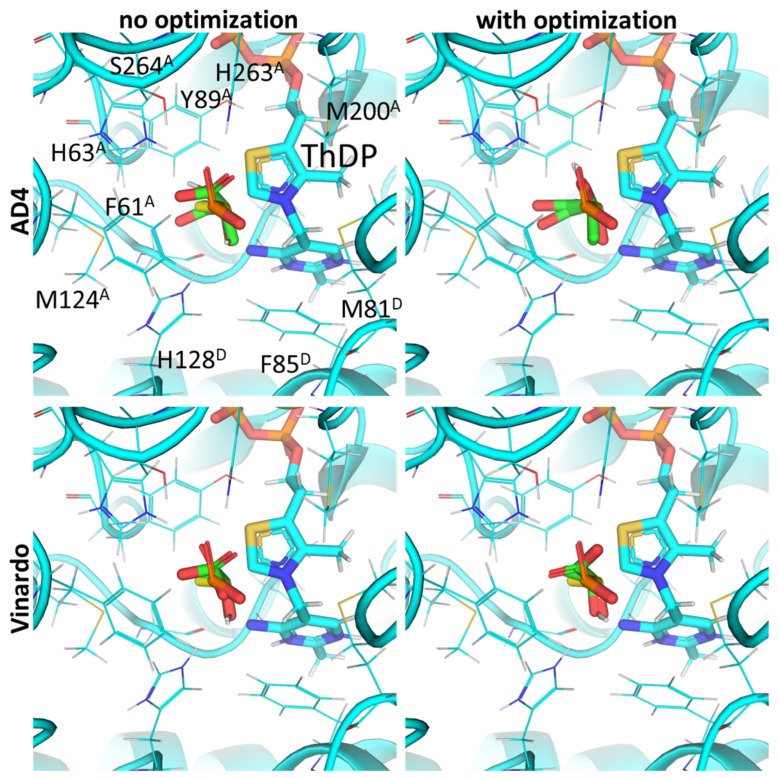
Comparison of optimal conformations upon covalent binding of pyruvate to the ThDP-PDH complex using various Gnina setups. Docking poses were obtained using AD4 or Vinardo scoring functions with and without UFF ligand optimization. Only conformations with the lowest binding energy in each run are shown. Atom color scheme is the same as in Figure 2A, except carbon atoms of docked pyruvate are colored in green, and carbon atoms of AcPH initially present in PDH structure—in yellow. Amino acids residues within 6Å of the docked pyruvate are labeled; superscripted letters indicate protein chains. Comparison to other substrates is presented in Figure S1.

The known substrates for PDH, pyruvate, 2-oxobutyrate, and glyoxylate resemble lactate, 2-hydroxybutyrate, and glycolate in their intermediate states, respectively (Figure 1, Table 1). Using the covalent docking approach, we compared the affinity of these oxo acids to ThDP within the PDH structure. The results suggest that AD4 scoring favors the binding of its bulkier 2-oxobutyrate (−13.21 kcal/mol) compared to the canonical substrate, pyruvate (−6.98 kcal/mol), or its even smaller homolog, glyoxylate (−9.14 kcal/mol) (Table 2). This seems to be due to local hydrophobicity within the PDH structure (Figure 4; Supplementary Figure S1). Noteworthily, it contradicts the published kinetic data, with the K_m_ for 2-oxobutyrate being twice as high as K_m_ for pyruvate [6]. However, the K_m_ values are linked to the reaction rate and thus dependent on large conformational changes and involve other subunits of PDH, transferring the acyl moiety to lipoamide (Figure 1), whereas our docking procedure is limited to modeling only one of the intermediates. Glyoxylate, on the other hand, was shown as an alternative substrate of plant chloroplastic PDH [50] and as an inhibitor of PDH from *E. coli* [51]. Its K_m_/K_i_ values are somewhat lower than K_m_ for pyruvate, which is generally similar to lower energies for glyoxylate vs. pyruvate estimated in silico with the AD4 function (Table 2). As for Vinardo scoring, it shows lower calculated binding energies for pyruvate vs. 2-oxobutyrate (Table 2), in accordance with the substrate specificity of the enzyme, however the calculated energies are higher, and the values are less robust. For example, pyruvate shows the highest affinity (−2.21 kcal/mol) compared to 2-oxobutyrate (−1.71 kcal/mol), similar to what is found in in vitro studies in the literature. Glyoxylate, however, is predicted to be a worse substrate (−1.57 kcal/mol) here (Table 2), contrary to the data demonstrated for plant PDH in vitro [50], but it has not been tested with mammalian enzymes. Nevertheless, as we observe later, both AD4 and Vinardo scoring functions can be used to predict which inhibitors will affect the cognate enzymes the most compared to other compounds.

Next, we estimate the relative affinity of pyruvate for synthetic substrate analogs, which can effectively inhibit PDH. Among them, AcPH is considered the most potent inhibitor of mammalian and bacterial PDH [52,53,54]. Gnina using AD4 scoring (Table 2) confirms this in silico with the lowest binding energy of −10.03 kcal/mol compared to other known inhibitors described in the literature. For example, AcMePH showed a 100-times higher K_i_ value for human PDH than AcPH [54], in accordance with its much higher predicted binding energy (−1.60 kcal/mol, Table 2).

The inhibitory effect of phosphonate analogs containing an additional oxygen atom is much weaker both in vitro and in silico. Methyl ester of acetyl phosphonate (AcPMe) showed 100 times weaker binding to human PDH than AcPH [52], similar to AcMePH [54]. However, AcPMe may be a weaker inhibitor than AcMePH due to its less noticeable time-dependent component in PDH inhibition than those of its phosphinate counterparts, AcPH and AcMePH [55]. Finally, AcP, which resembles pyruvate more than AcPMe, is a 100-times weaker PDH inhibitor, although this was shown on PDH isolated from plants only [56]. Moreover, although some indicate that AcP should be effective for PDH [57,58], there is evidence that AcP effect may be due to residual AcPMe present, as AcP is usually synthesized via AcPMe hydrolysis [56,59]. Here, covalent docking also shows stronger AcPMe binding when using both AD4 and Vinardo scoring, although the former results in positive binding energy for both compounds (Table 2). One of the mechanisms, which may result in preferential AcPMe vs. AcP binding is the electrostatic repulsion of additional negative charge (in AcP) from the C2-atom of ThDP. In pyruvate oxidase, which is a relatively similar ThDP-dependent enzyme, the K_i_ values for AcP and AcPMe are similar at pH 6 when the inhibitors should have a similar charge (−1), however K_i_ for AcPMe is 10 times lower than for AcP at pH 7, where AcP acquires a stronger (−2) charge [60].

Thus, since K_m_ values for the PDH substrates involve further transformation with other enzymes and substrates not taken into account by our covalent docking procedure, the efficiency of the two scoring functions is better revealed in the example of the PDH inhibitors, which do not depend on a further reaction cycle. Here, AD4 clearly shows better efficiency, as its predicted affinities for four pyruvate analogs (AcPH > AcMePH > AcPMe > AcP) are closer to the published in vitro studies. Ethyl ester of acetyl phosphonate (AcPEt) was reported to act as a slightly weaker PDH inhibitor, than AcPMe [55,61], however AD4 indicates the opposite (Table 2). Using Vinardo scoring, however, results in AcPMe and AcPEt as the strongest binding candidates; the rest of the pyruvate analogs follow the same trends but with little differences in calculated binding energies (AcPEt >> AcPMe >> AcPH > AcMePH > AcP; Table 2).

**Figure 5:**
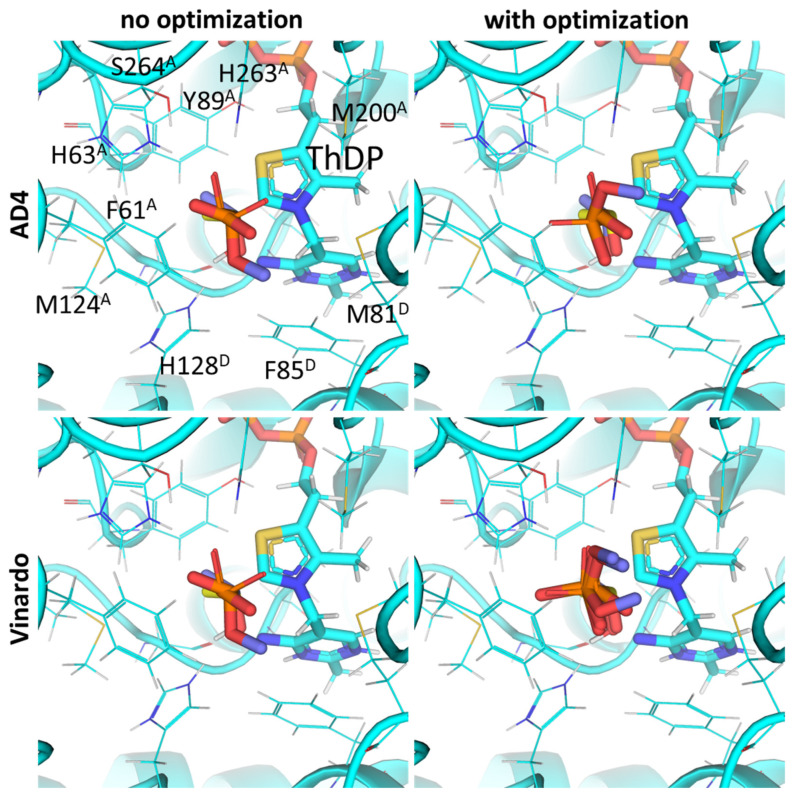
Comparison of optimal conformations upon covalent binding of synthetic pyruvate analog, AcPMe, to ThDP-PDH complex using various Gnina setups. Docking poses were obtained using AD4 or Vinardo scoring functions with and without UFF ligand optimization. Only conformations with the lowest binding energy in each run are shown. Atom color scheme and labels are the same as in Figure 4, except that the carbon atoms of docked AcPMe are slate colored. Amino acid residues within 6Å of the docked ligands are labeled; superscripted letters indicate protein chains. A comparison to other analogs is presented in Figure S2.

Thus, measurement of PDH activity in the presence of the known phosphonate substrate analogs in vitro and its simulation in silico both favor the binding of the esterified compounds (AcPMe and AcPEt vs. the de-esterified AcP), which, besides the negative charge of the C2 atom of ThDP, again, seems to be due to the hydrophobic residues near the ligand binding sites. The phosphono ester group-binding residues include Met200 and Phe61 from chain α (A) as well as Phe85, Met81, Ala36 and Tyr37 and from chain β’ (D) (Figure 5; Supplementary Figure S2). However, among the known substrate analogs for mammalian PDH, AD4 scoring only indicates their effective inhibition by AcPH and some by AcMePH, and not by the phosphonate pyruvate analogs (Table 2).

Fully esterified AcPMe_2_, however, is predicted to have an even higher affinity towards PDH when using AD4 scoring (Table 2), in accordance with some in vivo experiments [52,62], despite the absence of its inhibitory potential in vitro [52]. This discrepancy can be easily explained due to the rapid hydrolysis of AcPMe_2_ into acetic acid and dimethyl phosphite before further decomposing into metaphosphoric acid and methanol [63,64].

### 2.3. UFF Optimization Partially Improves Predictions Based on Vinardo but Not AD4 Scoring

Next, we determine whether utilizing the Universal Force Field (UFF) optimization of the bound ligand, which is available in Gnina, better distinguishes the bindings of strong (AcPH), moderate (AcMePH, AcPMe, AcPEt), and weak (AcP, AcPMe_2_) PDH inhibitors and their substrates (pyruvate, 2-oxobutyrate, glyoxylate). With the AD4 scoring function, the calculated relative affinity for pyruvate (−11.44 kcal/mol) is higher than that for glyoxylate (−9.26 kcal/mol) only when UFF optimization is applied (Table 2). On the other hand, the initial preference for pyruvate vs. glyoxylate is still observed in the Vinardo function after optimization (−2.73 vs. −1.61 kcal/mol), while the expected affinity for 2-oxobutyrate becomes the same as for pyruvate (−2.85 vs. −2.71 kcal/mol)—similarly to AD4 scoring without UFF optimization (Table 1).

Use of UFF optimization with synthetic pyruvate analogs still shows good RMSD values in the template AcPH (Figure 5). For the best conformation of AcPH itself, the optimization does not result in substantial changes in either RMSD or AD4 (1.49 vs. 1.47 Å), nor does it for Vinardo (1.37 vs. 1.47 Å) scoring functions (Supplementary Figure S2). Docking with optimization still results in AcPH showing the strongest binding to PDH for both the AD4 (−11.5 kcal/mol) and Vinardo (−2.83 kcal/mol) scoring functions (Table 2). However, AcP affinity is predicted to have higher inhibitory potential than AcPMe (−4.99 vs. 9.19 kcal/mol), i.e., contrary to the published in vitro data when using an AD4 function, and lower than AcPMe (−0.84 vs. −1.63 kcal/mol) when using Vinardo (Table 2). In addition, using UFF optimization with Vinardo increases the difference between AcP and AcPMe binding energies from 1.25 to almost 2 times (Table 2). Calculations for AcPMe_2_ binding are closer to AcP (Table 2), however their practical relevance is low due to the rapid hydrolysis of the AcPMe_2_ C-P bond, unlike AcP monoesters, which are stable in water [52].

Thus, based on the predicted affinities of the PDH substrates and inhibitors in a covalent docking setup and their comparison to in vitro data, we suggest AD4 scoring without using ligand UFF optimization as a better option for the determination of strong binding candidates such as AcPH. Vinardo scoring with ligand optimization can be used for the estimation of binding potency across less prominent candidates, such as AcP vs. its esters, AcPMe, AcPMe_2_, and AcPEt (Supplementary Figure S3). Nevertheless, all setups require many repeats, as even in the case of AcPH, there were several clusters of docked ligands localized in the PDH active site (Supplementary Figure S2), with only one of them corresponding to the “global” energy minimum of −10.03 kcal/mol.

### 2.4. Using Flexible Residue Setup Does Not Allow for Better Prediction of Ligand Binding to PDH

Finally, all pyruvate analogs were docked using a flexible residue setup. The results (shown in italics in Table 2) indicate that changes in bond angles for amino acid side chains give worse (i.e., further from published in vitro data) predictions in addition to much more time being required for a single docking run. For example, in all of the previously mentioned setups (AD4 vs. Vinardo scoring, UFF optimization vs. no optimization), AcMePH showed either a similar affinity to AcPH or AcP showed a similar or even higher affinity compared to AcPMe, contrary to previous in vitro studies (see Section 2.2). This discrepancy is due to the relative positions of the bound ligands and amino acid side chains being highly dependent on the initial seed value. This results in higher variations in docking run outputs and thus probably requires much more computation-heavy repeats to find the minimal energy conformation.

### 2.5. BCDH Shows Preference in Binding of Esterified vs. Non-Esterified Ligands Similarly to PDH

The specificity of BCDH towards potential ligands is much wider, including the PDH substrates, pyruvate, and 2-oxobutyrate, but also various branched-chain 2-oxo acids [6,65]. The specific BCDH substrates are transamination products for L-valine, L-leucine, and L-isoleucine, and probably its diastereomer, L-alloisoleucine [66], known as 2-oxoisovalerate, 2-oxoisocaproate, (S)-3-methyl-2-oxovalerate, and (R)-3-methyl-2-oxovalerate (Table 3).

**Figure 6:**
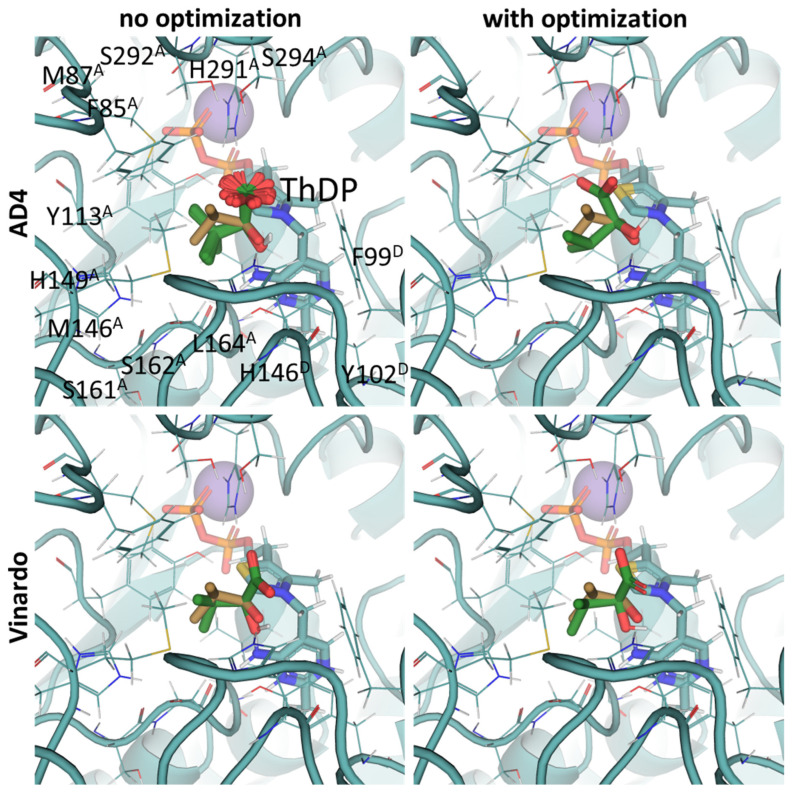
Comparison of optimal conformations upon covalent binding of 2-oxoisovalerate to ThDP-BCDH complex using various Gnina setups. Docking poses were obtained using AD4 or Vinardo scoring functions with and without UFF ligand optimization. Only conformations with the lowest binding energy in each run are shown. Carbon atoms of the BCDH protein template from PDB ID: 2J9F (including ThDP and nearby amino acid residues) are colored in marine; carbon atoms of the docked 2-oxoisovalerate are in green, and carbon atoms of the decarboxylated 2-oxoisovalerate initially present in the BCDH structure are sand-colored; non-carbon atoms are colored according to standard color scheme. Amino acids residues within 6Å of the docked ligands are labeled; superscripted letters indicate protein chains. Comparison to other substrates is presented in Figure S4.

Our docking results with BCDH and AD4 scoring functions show the accurate placement of 2-oxoisovalerate (RMSD of 1.49Å from template 2-oxoisovalerate; Figure 6) and confirm that the branched-chain 2-oxo acids should have higher affinity with BCDH than pyruvate and 2-oxobutyrate (Table 2). This, again, seems to be due to multiple hydrophobic residues binding the branched-chain aliphatic radical, such as Phe85, Met87, Tyr113, Met146, and Leu164 from chain α (A) (Figure 6, Supplementary Figure S4). However, the biologically relevant S-enantiomer of 3-methyl-2-oxovalerate seems to bind weaker than its corresponding R-enantiomer or similar analogs of valine and leucine (Table 2, Supplementary Figure S4). Existing enzymological studies mention that BCDH’s affinity for these substrates is an increasing in order of pyruvate < 2-oxobutyrate < 2-oxoisovalerate < 2-oxoisocaproate ≈ 2-oxo-3-methylvalerate [6,65,67], but the latter always seems to be used as a racemic mixture and the kinetics data should be compared with the average predicted energy for two enantiomers.

However, similarly to PDH substrates, the existing scoring functions do not allow an accurate estimation of BCDH substrate specificity, i.e., both AD4 and Vinardo functions do not show results identical to in vitro studies, as docking predicts stronger binding of shorter substrates, i.e., pyruvate or 2-oxoisovalerate, depending on the scoring and whether UFF optimization is used (Table 2). The relative predicted affinities closest to the relative K_m_ values are achieved when using AD4 scoring with UFF optimization (Table 2) which also decreases RMSD values slightly (1.49 vs. 1.37 Å), similarly to Vinardo (1.52 vs. 1.46 Å). Nevertheless, as already mentioned, K_m_ values in the literature do not simply indicate the initial binding and formation of the intermediate, but also the efficiency of further substrate transformations, such as transfer to lipoamide.

**Figure 7:**
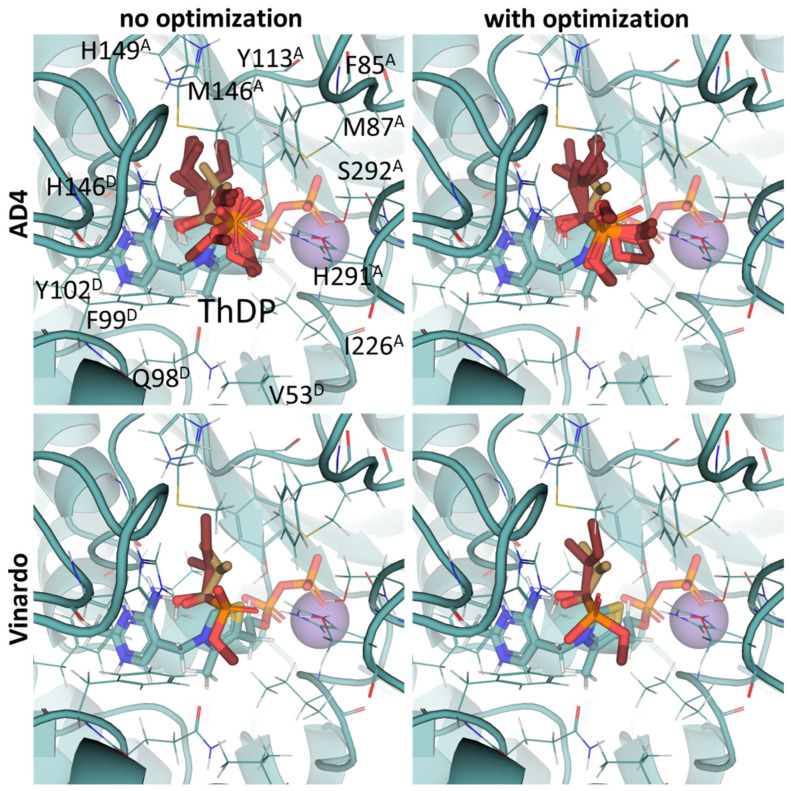
Comparison of optimal conformations upon covalent binding of synthetic (S)-3-methyl-2-oxovalerate analog, (S)-MBP, to ThDP-BCDH complex using various Gnina setups. Docking poses were obtained using AD4 or Vinardo scoring functions with and without UFF ligand optimization. Only conformations with the lowest binding energy in each run are shown. Atom color scheme is the same as in Figure 6, except the carbon atoms of docked (S)-MBP are firebrick-colored. Amino acids residues within 6Å of the docked ligands are labeled; superscripted letters indicate protein chains. Comparison to other analogs is presented in Figure S5.

Our docking procedure seems better for the estimation of the inhibitory potencies of 2-oxo acid analogs, than for the substrate binding of 2-oxo acids themselves. Regarding the synthetic analogs of branched-chain 2-oxo acids as potential BCDH inhibitors, only phosphonate analogs of isoleucine/alloisoleucine, 2-methylbutyryl phosphonate methyl (MBPMe), and diethyl (MBPEt_2_) esters have been described in one of our previous papers [68], where racemic mixtures of (R)- and (S)-2-methylbutyryl phosphonates were synthesized. Any of the four used docking setups (AD4 or Vinardo scoring, both with and without UFF optimization) indicate the stronger binding of partially esterified compounds (MBPMe) vs. non-esterified (MBP) or fully esterified (MBPMe_2_, MBPEt_2_) ones, with at least twice-lower estimated binding energies for MBPMe vs. MBP (Table 2). These results are similar to PDH ligands (see Section 2.2 and Section 2.3) and, as in the case of PDH, this also seems to be mediated by the local hydrophobic regions in the BCDH structure binding the ester moieties (Figure 7). However, the difference in the predicted affinities of MBP vs. MBPMe in BCDH seems to be higher than in those of AcP vs. AcPMe in PDH, probably due to the larger number of hydrophobic residues in the active site of the former enzyme, including Met146, Phe85, Met87, and Ile226 in chain α (A) and Val53, Phe54, Phe99, and Tyr102 in chain β’ (D) (Figure 7, Supplementary Figure S5). The difference in predicted affinities is even higher for the esterified phosphonate analogs of other BCDH substrates, isobutyryl phosphonate, a 2-oxoisovalerate analog, and isovaleryl phosphonate, a 2-oxoisocaproate analog (Table 2), which, however, have not been synthesized and characterized yet.

### 2.6. Docking of ω-Carboxylated Ligands Favors Binding of De-Esterified Substrate Analogs

**Figure 8:**
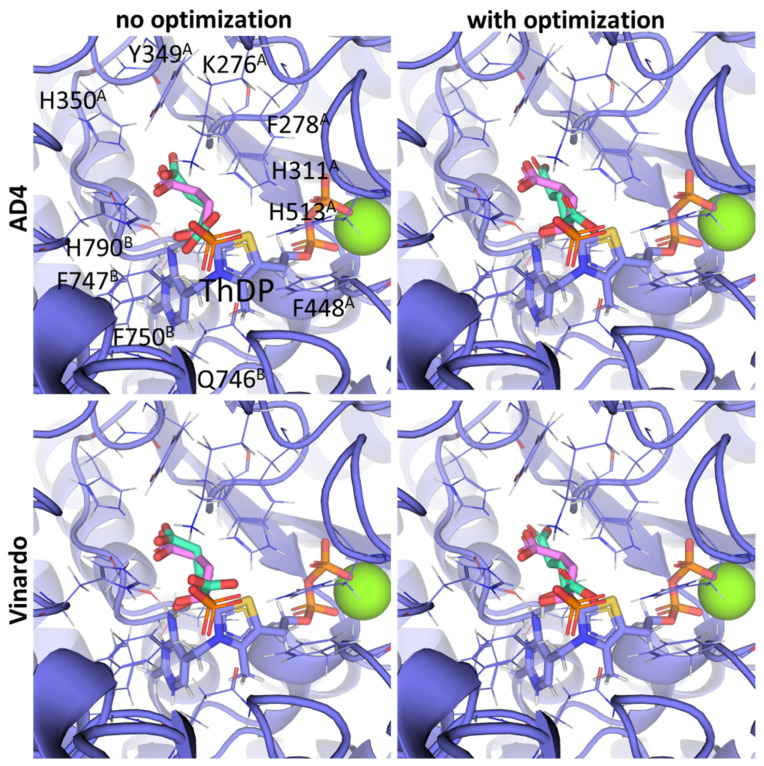
Comparison of optimal conformations upon covalent binding of 2-oxoglutarate to ThDP-OGDH complex using various Gnina setups. Docking poses were obtained using AD4 or Vinardo scoring functions with and without UFF ligand optimization. Only conformations with the lowest binding energy in each run are shown. Carbon atoms of OGDH protein template homology modeled from PDB ID: 2YID and 6R29 (including ThDP and nearby amino acid residues) are indigo-colored; carbon atoms of docked 2-oxoglutarate are in aquamarine, and carbon atoms of SP initially present in the OGDH structure are lilac-colored; non-carbon atoms are colored according to standard color scheme. Amino acids residues within 6Å of the docked ligands are labeled; superscripted letters indicate protein chains. Comparison to other substrates is presented in Figure S6.

Since no crystal structure of mammalian OGDH (either with or without 2-oxo ligands) is available in PDB except for two cryo-EM structures with the wrong ThDP placement or a ThDP analog instead of ThDP [69,70], its affinity towards potential substrates and inhibitors was studied using a homology-modeled structure from bacterial OGDH (see Section 4.2). The resulting template structure shows similar predicted affinities for the two known OGDH substrates, 2-oxoglutarate and 2-oxoadipate (Table 4), with minor preference towards the latter (Supplementary Figure S6, Table 2). The obtained results were similar for both scoring functions and were not affected by the UFF optimization of the ligand (Figure 8, Table 2). However, those do not agree with in vitro studies with recombinant and isolated enzymes, where 2-oxoadipate is shown to be a much weaker substrate [14,71]. This also discourages the use of our docking setups for testing the substrate specificity of the enzymes. Nevertheless, although the 2-oxoadipate shows lower activity and a higher K_m_ value, its binding to OGDH results in the irreversible inactivation of the enzyme [72,73,74] due to weaker involvement in the further steps of oxidative decarboxylation reactions. Instead, 2-oxoadipate oxidative decarboxylation by OGDH results in excessive ROS formation [71], which may additionally inhibit the corresponding enzymes via oxidative damage [4,75].

Unlike the experiments with isolated PDH and BCDH, the kinetic analysis of OGDH activity in the presence of its inhibitors showed a similar potency for 2-oxoglutarate analog, succinyl phosphonate (SP), vs. its phosphono-esters (Supplementary Figure S7), the most mentioned of which in the literature is phosphonoethylated SP derivative, PESP (Table 2) [76,77]. In some experiments, SP has even acted as stronger inhibitor than PESP [78], although the relative effects heavily depend on the preincubation of OGDH with inhibitors [76]. Indeed, the estimated affinities of SP and PESP towards OGDH are the same, according to all four docking setups (Table 2). At the same time, the predicted binding of the phosphonomethylated derivative of SP (PMSP) are weaker than those for both SP and PESP and is in agreement with its lower inhibitory potential in vitro [73,79,80]. Phosphonodimethylated derivative (PDSP), in addition to being a weaker ligand in both in vitro [79] and in silico (Table 2) experiments, is likely unstable in aqueous solutions anyway [26], similarly to AcPMe_2_.

**Figure 9:**
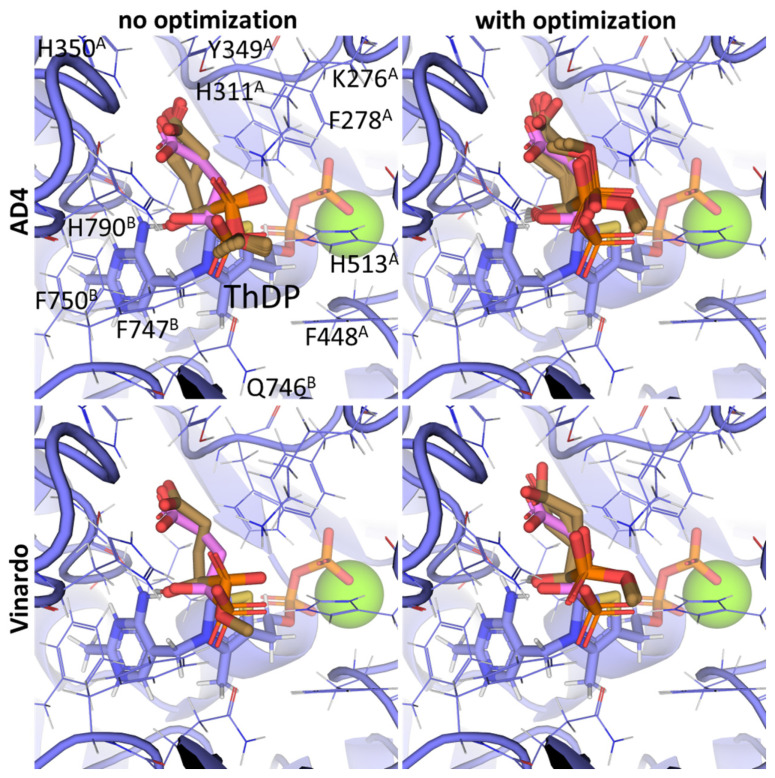
Comparison of the optimal conformations upon covalent binding of the 2-oxoglutarate phosphonate analog, PMSP, to the ThDP-OGDH complex using various Gnina setups. The docking poses were obtained using the AD4 or Vinardo scoring functions with and without UFF ligand optimization. Only the conformations with the lowest binding energy in each run are shown. The atom color scheme is the same as in Figure 8, except that the carbon atoms of docked PMSP are olive-colored. Amino acid residues within 6Å of the docked ligands are labeled; superscripted letters indicate protein chains. A comparison to other analogs is presented in Figure S7.

Other SP derivatives, the diethyl (DESP) and triethyl (TESP) esters of SP, are bulkier and show lower predicted affinities in accordance with the in vitro experiments, where they did not inhibit OGDH at all [77]. This similarity in the binding of esterified vs. non-esterified phosphonates, or even a preference towards the non-esterified phosphonates in OGDH, corresponds to a lower number of hydrophobic residues in the cognate areas of the enzyme structure compared to PDH and BCDH, and a more-confined substrate-binding pocket. The pocket includes some hydrophobic residues, like Phe278 and Phe48 from chain α (A) and Phe747 and Phe750 from chain α′ (B), but many more hydrophilic ones, resulting in steric clashes of esterified SP derivatives with both polar and non-polar OGDH atoms (Figure 9). The carboxyethyl ester of SP (CESP), however, despite similar inhibitory potential in mammalian OGDH as SP in vitro [77], shows the lowest predicted binding energies in our studies (Table 2). Finally, glutaryl phosphonate (GP), a longer SP homolog, is predicted to bind to OGDH similarly to SP (Table 2, Supplementary Figure S7) and shows similar inhibitory potential to SP in in vitro studies [14,26].

## 3. Discussion

### 3.1. Using Covalent vs. Conventional Docking for Modeling Ligand Binding to ThDP-Dependent Enzymes

The existing approaches for docking into the active sites of ThDP-dependent enzymes mainly relies on one of three designs. The first one uses a conventional (i.e., non-covalent) docking setup, as shown, for example, for bacterial PDH [35], bacterial 2-oxoglutarate decarboxylase [81], as well as transketolase [36,82,83] and related transketolase-like carboligases [38,84,85], which prevent the correct placing of ligands near ThDP and the identification of interacting amino acid residues.

The second one docks modified ThDP structures, replacing the ThDP initially present in the structures. It was first utilized for studying various thiamine antagonists, such as oxythiamine diphosphate and triazole-ThDP, binding to the active sites of human PDH, OGDH, and related enzymes, yeast pyruvate decarboxylase and bacterial pyruvate oxidase [86]. Later the approach was modified to include acyl-adducts of the selected antagonists, deaza-ThDP and furan-ThDP [87,88]. Others replaced ThDP with pyrimidine hydrazone, pyrimidine urea, and pyrimidine clomazone derivatives as well as other multiple thiamine antivitamins [37,89,90,91,92,93,94]. The same strategy of ThDP replacement with antagonists or covalent ThDP-ligand adducts is described in many papers for bacterial, plant, and protozooan TKT [95,96,97,98,99,100], and related 1-deoxy-D-xylulose-5-phosphate synthase [38,101] and acetohydroxyacid synthase (AHAS) [102,103,104]. In addition, some have described the conventional docking of the second/acceptor substrates (“Z” in Figure 1) into TKT near ThDP [36,105] or the dihydroxyethyl-ThDP adduct [36]. Nevertheless, none of these studies actually discusses the relative affinities of several “donor” (“Oxo-substrate” in Figure 1) ligands that covalently bind to ThDP and thus initiate the ThDP-dependent reaction or inhibit the enzyme. Moreover, the poses of the docked ThDP adducts should be mainly determined by bulky ThDP moieties, not the attached substrates/inhibitors.

The third approach utilizes the alignment of target proteins in conjunction with ThDP or its adducts and manual replacement of the parts corresponding to oxo-ligands with dictionaries of bulkier ligands [26]. Although this approach visualizes interactions between the ligands and protein residues and is used with many other proteins [106], its ligand-binding affinities cannot be properly estimated.

We have evaluated the fourth approach in this study based on the existing target structures: choosing one of the available setups resulting in the most accurate conformation of bound ligands and in the predictions closest to in vitro studies. Similarly to other covalent docking algorithms, this approach does not model the covalent binding itself, only estimates the affinity of some reaction intermediates. However, despite struggling with substrate kinetics, we have demonstrated an opportunity for the prediction of the relative affinities of several ThDP-dependent enzyme inhibitors (Table 2).

The approach we have evaluated based on the example of ThDP could be expanded to model the binding of ligands to other enzymes with different cofactors. Potential targets include, but are not limited to, ubiquitous enzyme ligands such as pyridoxal-5′-phosphate (PLP) [107,108,109,110,111,112], NAD/NADP [113,114], FAD [115] and 5′-deoxyadenosylcobalamin [116]. For example, D-cycloserine is a well-known covalent inhibitor used for the study of several PLP-dependent enzymes, including in the search for new drugs [107,108]. Similar clinically relevant inhibitors are gabaculine and vigabatrin, inactivating GABA transaminase [109,110], and elfornitine, an inhibitor of ornithine decarboxylase [111,112]. Although even more advanced computational approaches are presented in the literature, for both ThDP-dependent (see Section 3.3) and PLP-dependent enzymes [117], almost none of the studies compare the affinities of ≥2 ligands binding to the same active site.

### 3.2. Influence of Hydrophobic Regions in Substrate Binding Sites of PDH and BCDH, but Not OGDH, on Enzyme Substrate and Inhibitor Specificity

The data obtained in this paper indicate the presence of several hydrophobic pockets in the substrate-binding sites of PDH and BCDH (Figure 4, Figure 5, Figure 6 and Figure 7). These pockets seem to be responsible for the binding of bulkier, esterified ligands like AcPMe and MBPMe (Table 2), which is confirmed by published in vitro studies with AcPMe. One of the two hydrophobic pockets in the human PDH structure was also found during the analysis of the PDB ID:6CFO structure with docked acylated furan-ThDP derivatives, suggesting that the binding of bulkier ThDP analogs may mimic the binding of another PDH substrate, lipoamide, which is highly hydrophobic [88]. Here we confirm the presence of this pocket, responsible for the lower calculated affinities of pyruvate vs. 2-oxobutyrate, for example, but we also add another, which is responsible for the tighter binding of esterified ligands (AcPMe, AcPMe_2_ and AcPEt vs. AcP; Figure 5, Table 2). The presence of the same pockets in BCDH is what mediates its specificity towards the branched-chain 2-oxo acids vs. pyruvate [118] and should also favor the binding of esterified oxophosphonates (Table 2). OGDH, on the other hand, has significantly less hydrophobic residues near its phosphonate moiety (Figure 8 and Figure 9), resulting in similar predicted affinities for SP and its phosphono-esterified derivatives, PMSP and PESP (Table 2). The presence of a hydrophobic lipoamide-binding site nearby may, however, mediate its higher predicted affinities for carboxy-esterified compounds, like CESP (Table 2).

All of this indicates the importance of the structural analysis of target proteins for developing new drugs. That is, for some ligands, such as AcPMe, partial esterification increases its inhibitory potential towards PDH, while for others, like OGDH inhibitor SP, a more polar substrate-binding site promotes stronger binding for non-esterified inhibitors. However, since OGDH should bind the same hydrophobic lipoamide residue acceptor substrate, some of the bulkier substrates, like 2-oxoadipate, may act as inhibitors, partially occupying the corresponding pocket. This would explain the irreversible inactivation of the 2-oxoadipate dehydrogenase reaction when catalyzed by OGDH [72,73,74] and not its isoenzyme, DHTKD1, with a more spacious active site [26,119]. Thus, DHTKD1 or DHTKD1-mimicking OGDH mutants instead accept not only 2-oxoadipate, but even bulkier 2-oxopimelate, 2-oxosuberate, or even the branched-chain 2-oxo acids [12,120,121]. The same is true for the inhibitors: DHTKD1 may easily accept GP and even bulkier adipoyl phosphonate and pimeloyl phosphonate as inhibitors, while the OGDH-specific SP does not affect a DHTKD1-catalyzed reaction with 2-oxoadipate as a substrate [12]. Analysis of the preference of the DHTKD1 isoenzyme for esterified vs. non-esterified phosphonate inhibitors has not yet been tested in vitro though.

Thus, our results underline the importance of ligand modifications, including ester groups, for the search for stronger and even more specific inhibitors of ThDP-dependent enzymes, among others. Aside from the different preferences of OGDH and its paralog DHTKD1 towards 2-oxoglutarate and SP homologs, this is exemplified by acylated deaza-ThDP derivatives, which, depending on the exact acyl moiety, showed a preference towards either pyruvate decarboxylase or a related enzyme, pyruvate oxidase [60,87].

### 3.3. Further Directions

A similar approach can be applied to other ThDP-dependent enzymes. First, the OGDH isoenzymes, encoded by paralogous *OGDHL* and *DHTKD1* genes, has drawn much attention in medicine. The first, expressed mainly in the brain, apparently has a higher affinity for 2-oxoglutarate than the canonical OGDH, but is inactive as a recombinant protein [122]. The second, expressed mostly in the liver and kidneys [14] is known to be specific towards bulkier substrates and inhibitors (see Section 3.2). However, despite several crystallographic structures of DHTKD1 being obtained recently by the Yue and Lazarus groups [12,123], no structures of mammalian OGDH or its OGDHL isoenzyme have been deposited to PDB, except for two cryo-EM models with certain disadvantages (see Section 4.2). As a result, we have had to rely on the homology model of mammalian OGDH based on the bacterial structure, but ideally, these experiments should be repeated with either a crystallographic or a better cryo-EM mammalian OGDH in conjunction with ThDP and either SP/GP or 2-oxoglutarate/2-oxoadipate as an initial protein template.

Taking into account the results obtained here, it would be very interesting to analyze the carboligase side reactions catalyzed by DHTKD1, which are significant for the enantiomeric synthesis of various ketol compounds [121,124]. Interestingly, DHTKD1-catalyzed carboligase reactions have been shown to result in different chirality when 2-oxoglutarate and 2-oxoadipate are used [121]. Thus, the modeling of adducts with both (R)- and (S)-hydroxyderivatives should provide new data on the potential molecular mechanisms of such intriguing differences.

In addition to the three paralogs of *OGDH*, there are also three paralogs of the *TKT* gene in the human genome. The canonical, ubiquitously expressed TKT is almost substituted by TKTL1 and TKTL2 isoenzymes in germline cells according to available single-cell data [125,126]. Despite a strong germline-specific expression pattern, TKTL1 also has minor expressions in different organs, including the brain, where the enzyme is primarily present in the parastriate area of the occipital cortex [127], and its function affects the formation of neuroprogenitor cells [128]. TKTL2, on the other hand, is significantly expressed only during the later stages of spermatozoa maturation [125,126]. Being an enzyme that is important for cell biosynthesis, TKT, as well as both the TKTL1 and TKTL2 isoenzymes, have turned out to be important prognostic markers and potential regulatory targets in various cancer types (reviewed in [129]). Thus, taking into account the role of transketolase, a great number of its substrates, and also lack of resolved protein structures of non-canonical isoenzymes, analysis of transketolase’s interactions with its substrates deserves a separate study. However, a brief test of our proposed docking algorithm on human TKT and its substrate, D-xylulose-5-phosphate-derivative (D-arabitol-5-phosphate), supports its broader applicability. The results show reliable RMSD values of 3.00–3.12 Å, depending on the optimization and scoring functions (Supplementary Figure S8), and decent binding energies (Table 2).

As for the final mammalian ThDP-dependent enzyme, HACL, there are peroxisomal HACL1- and EPS-localized membrane-bound HACL2 (*HACL1* and *ILVBL* genes, respectively). Both catalyze carboligase-like reactions in the fatty acid α-oxidation pathway [130] with seemingly very close substrate specificities. Dysfunction in both isoforms is linked to the same rare pathology, Refsum disease [21]. No structure of mammalian HACL has been resolved for the moment, although structural data on bacterial or fungal enzymes, in conjunction with ThDP and oxo-ligands, have recently been obtained [130,131]. Covalent molecular docking can thus be a useful instrument for developing a better understanding of substrate specificity and the function of the two human HACL isoenzymes. However, a deeper understanding of the catalytic mechanism of this enzyme sub-family is still required [131].

Additional progress in the modeling of covalently bound complexes may come from the application of neural network algorithms, which could be utilized in some of the Gnina setups. However, both Gnina developers [44,45] and independent researchers [28] have discussed the lack of training data regarding covalent ligand–protein complexes. Thus, a high level of expertise in the structure and functioning of specific protein families (or, at least, validated protein structure model(s) and in vitro assays) is required initially to check the accuracy of covalent docking predictions. Otherwise, suboptimal parameters may undermine the efficiency of the conformational search and affinity predictions [28,44,45,132,133].

However, multiple researchers are now using molecular dynamics approaches to better characterize the parameters of ligand binding, such as retention time in the protein site. Nevertheless, most of the advanced computational techniques used for ThDP-dependent enzymes either just model the dynamics of the substrate binding near ThDP [134,135] or of various reaction intermediates covalently bound to the ThDP molecule alone [136,137] or to the ThDP within the structures of PDH, AHAS, TKT, or the related benzoylformate decarboxylase [138,139,140,141,142,143,144]. In the case of AHAS, however, the formation of the next intermediate has also been modeled, adding an acceptor substrate (“third intermediate” in Figure 1) and even the product release from ThDP in the carboligation reaction [140,141,142], but not the formation of the covalent bonds themselves. However, none of the mentioned studies have estimates the binding energies of ≥2 ligands or their stability as ThDP adducts, and thus none of the studies has compared their predicted affinities with actual K_m_ values or inhibitory potentials.

In addition, the modeling of the slow formation of the covalent bond between ThDP and the ligands may be even more important, especially for partially irreversible inhibitors, such as AcPH on PDH [52,53,55], or 2-oxoadipate on OGDH [72,73,74]. Such modeling, however, requires additional tools like QM/MM, with alchemical calculations, and even stronger expertise in chemical biology. These even-more-advanced computational techniques are indeed capable of simulating the enzymatic reactions when passing through energy barriers. To our knowledge, they have been applied to one human ThDP-dependent enzyme, TKT [145,146], bacterial PDH [147], and multiple unique bacterial enzymes, namely, phosphoketolase [148], benzoylformate decarboxylase [149,150], 2-succinyl-5-enolpyruvyl-6-hydroxy-3-cyclohexene-1-carboxylate synthase, or MenD [151], AHAS [152], oxalyl-CoA decarboxylase [153], pyruvate decarboxylase [154], benzaldehyde lyase [155], cyclohexane-1,2-dione hydrolase [156], and pyruvate–ferredoxin oxidoreductase [157,158]. But even then, although the methodology is apparently widespread, neither of the developed computational models compares the reaction flows or inhibition potentials, even for two ligands, except for one paper on pyruvate–ferredoxin oxidoreductase [158], estimating the reaction energy barriers for six potential 2-oxo acid substrates. Moreover, none of these advanced tools have been used for the investigation of pseudo-irreversible inhibitors, e.g., phosphonate and phosphinate substrate analogs. And although this approach is desirable for mammalian ThDP-dependent enzymes, such as PDH, BCDH, OGDH, TKT, and HACL with their isoenzymes, it essentially requires brute-forcing multiple parameters to reliably compute the energy profiles for a reaction with at least two different substrates or inactivation by at least two different inhibitors. Thus, the desired analysis may not even be available in the next few years. Machine learning predictions [159,160] could facilitate such studies, although those still require thousands of the existing structures of enzyme–intermediate complexes, which does not appear to be resolved yet.

Thus, even a computationally simple model, like the one presented here, can predict the affinities of various inhibitors of ThDP-dependent enzymes, which has been confirmed with the existing experimental data, whereas more advanced approaches have not yet been used for the relative estimation of inhibitory potencies towards the studied enzymes.

## 4. Materials and Methods

**Figure 10:**
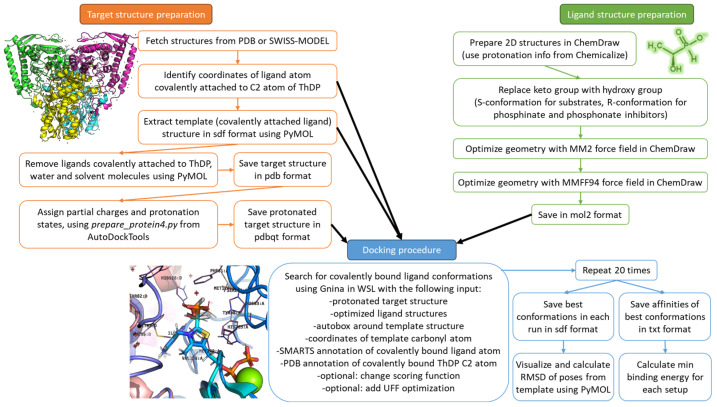
Schematic workflow of the docking algorithm used in the study. Green, orange and blue colors correspond to ligand structures preparation, target structures preparation and docking procedure, described in Section 4.1, Section 4.2 and Section 4.3, respectively.

The general workflow of the docking procedure as well as target and ligand preparations are presented in Figure 10 and described in the corresponding subsections below.

### 4.1. Ligand Structures Preparation

Structures of docked ligands were prepared as (R)- or (S)-hydroxy-derivatives (Table 1, Table 3 and Table 4) in Chem3D v.18.0 (PerkinElmer Inc., Waltham, MA, USA), and energy was minimized with built-in MM2 and MMFF94 force fields. The negative charges to the carboxyl, phosphinate, or phosphonate groups were assigned according to the Chemicalize database (https://chemicalize.com/, accessed on 11 December, 2024, developed by ChemAxon, Budapest, Hungary) at pH 7.5. The minimized structures were saved in mol2 format.

### 4.2. Target Structures Preparation

Structures of human PDH (PDB ID: 6CFO, [39]), human BCDH (PDB ID: 2J9F, [161]), and human TKT (PDB ID: 4KXW, [162]) containing ThDP covalently bound to their corresponding oxo-ligands (AcPH for PDH, decarboxylated 2-oxoisovalerate for BCDH, D-xylulose-5-phosphate for TKT) were downloaded from PDB using an open-source build of PyMOL v.3.0 (Schrödinger, Inc., New York, NY, USA) within the open-source JupyterLab shell (https://github.com/jupyterlab/jupyterlab, accessed on 30 June 2020, supported by Linux Foundation, San Francisco, CA, USA). Unfortunately, the existing mammalian OGDH cryo-EM structures in PDB either contained errors in their ThDP atom positioning (PDB ID: 7WGR, [69]) or a ThDP analog (PDB ID: 8I0K, [70]), and their resolution was relatively low for docking (both about 2.9Å). As a result, the rat OGDH template from the previous study [26] was used instead. The structure was homology-modeled from the mycobacterial OGDH (PDB ID: 2YID, [163]) using the SWISS-MODEL Web server [164], and the ThDP-OG adduct was replaced with the ThDP-SP adduct from another mycobacterial OGDH structure (PDB ID: 6R29, [165]) by structural alignment in PyMOL v.3.0 (Schrödinger, Inc., New York, NY, USA).

The ligands covalently bound to ThDP were removed together with water and other non-bound molecules (such as ethylene glycol) from the PDH, BCDH, TKT, and OGDH structures using PyMOL. The hydrogen atoms as well as partial atom charges were added to the resulting protein–ThDP structures using the prepare_protein4.py script with a built-in Python 2.7 shell supplied by AutoDockTools4 (Scripps Research, San Diego, CA, USA) software [166].

### 4.3. Docking Procedure

The ligand structures were docked into the protein targets using Gnina v. 1.3 [44] in the WSL environment with the following parameters:

- C2 atom of ThDP must be bound to a specific hydroxylated carbon atom (corresponding to a reactive carbonyl atom) of a ligand, indicated by ‘[C;H2]([O])’ (glyoxylate), ‘[C@H]([O;H1])([C;H2][O;H1])’ (xylulose-5-phosphate) or ‘[C@H]([O])’ (all the rest ligands) SMARTS patterns;
- docking box is autogenerated using bound ligand coordinates from the initial protein structure with default buffer space;
- reactive atom of a ligand should be fixed in specified coordinates, chosen from the initially bound ligand from protein structure template;
- hydrogen atoms should not be added, as those were added previously to both ligand and protein structures;
- exhaustiveness parameter should be as high as possible;
- different scoring functions (--scoring <name>), recalculations with convolutional neural networks (--cnn_scoring <name>) and UFF optimizations of bond angles and lengths of bound ligands (--covalent_optimize_lig) were used in some of the runs as specified in the text;
- when flexible side chain setup is used, only residues within 5.5 Å from an initially bound template were chosen (--flexdist 5.5); in that case ThDP itself is not considered as flexible residue. Note, this can be changed via exact specification of flexible residues (--flexres A:61,A:63,A:89,A:124,A:138,A:200,A:263,A:264,D:37,D:81,D:85,D:128,E:401);
- when flexible side chain setup is used, the high number of ligand and amino acid conformations were clustered based on RMSD values (--min_rmsd_filter 2).

All docking runs were repeated at least 20 times to ensure consistency in their optimal positioning and minimal binding energies. A typical script in WSL should look like this:


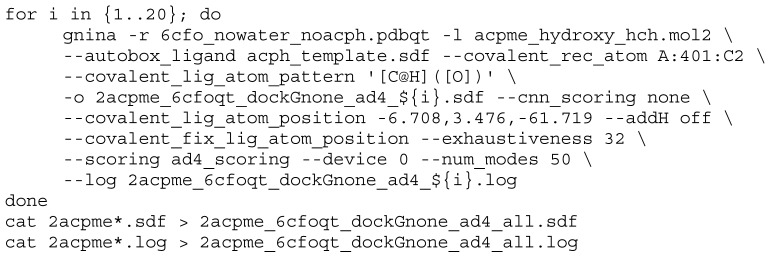


The minimal binding energies for all runs are presented in the corresponding sections of the text. The resulting ligand conformations were visualized in PyMOL.

## 5. Conclusions

Here we propose an algorithm for the covalent docking of substrates and inhibitors that bind to ThDP in the active sites of ThDP-dependent enzymes. The algorithm is evaluated and compared to the published in vitro experimental data on the members of mammalian 2-oxo acid dehydrogenases family—PDH, BCDH and OGDH. We believe the approach is not limited to ThDP-dependent enzymes and should also be applied to other ligands that form covalent bonds with non-protein cofactors, such as some inhibitors of pyridoxal-5′-phosphate-, NAD/NADP-, cobalamin-, or flavine-dependent enzymes.

## Figures and Tables

**Table 1:** Structures of the PDH ligands and their hydroxy-variants, known from the literature and used in the docking simulations in this study.

Type of Ligand	Actual Ligand	Ligand for Docking in Gnina
Substrates	Pyruvate 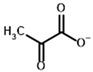	(S)-Lactate 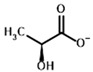
	2-Oxobutyrate 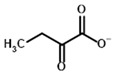	(S)-2-hydroxybutyrate 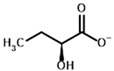
	Glyoxylate 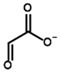	Glycolate 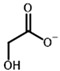
Inhibitors	Acetyl phosphinate (AcPH) 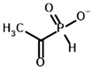	[(R)-1-Hydroxyethyl] phosphinate 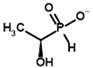
	Acetyl (methyl) phosphinate (AcMePH) 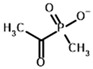	[(R)-1-Hydroxyethyl] (methyl) phosphinate 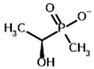
	Acetyl phosphonate (AcP) 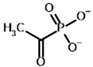	[(R)-1-Hydroxyethyl] phosphonate 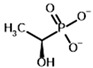
	Methyl acetyl phosphonate (AcPMe) 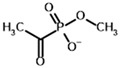	Methyl [(R)-1-hydroxyethyl] phosphonate 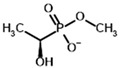
	Dimethyl acetyl phosphonate (AcPMe_2_) 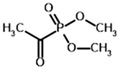	Dimethyl [(R)-1-hydroxyethyl] phosphonate 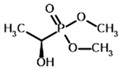
	Ethyl acetyl phosphonate (AcPEt) 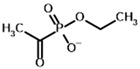	Ethyl [(R)-1-hydroxyethyl] phosphonate 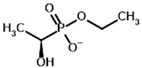

**Table 2:** Minimal binding energies for PDH, BCDH, OGDH, and TKT ligands across 20 runs in Gnina with and without UFF optimization of the bound ligand, using AD4 and Vinardo scoring functions. Affinities for PDH ligands also include those obtained with flexible side chains setup, shown in grey italics.

Enzyme Target	Type of Ligand	Name of the Ligand (Oxo-From)	Calculated Affinities, kcal/mol			
			Without Ligand Optimization		With Ligand Optimization	
			AD4 Scoring	Vinardo	AD4 Scoring	Vinardo
Pyruvate dehydrogenase (PDH, EC 1.2.4.1)	Substrates	Pyruvate *with flexible residues*	−6.98 *−12.44*	−2.21 *−2.84*	−11.44 *−16.35*	−2.73 *−3.09*
		2-Oxobutyrate *with flexible residues*	−13.21 *−16.40*	−1.71 *−2.86*	−16.17 *−18.14*	−2.85 *−3.80*
		Glyoxylate *with flexible residues*	−9.14 *−12.13*	−1.57 *−2.55*	−9.26 *−13.49*	−1.61 *−2.69*
	Inhibitors	Acetyl phosphinate (AcPH) *with flexible residues*	−10.03 *−11.75*	−2.28 *−2.74*	−11.50 *−17.19*	−2.83 *−3.39*
		Acetyl (methyl) phosphinate (AcMePH) *with flexible residues*	−1.60 *−11.69*	−2.22 *−2.50*	−4.44 *−14.08*	−2.48 *−3.57*
		Acetyl phosphonate (AcP) *with flexible residues*	9.28 *−10.98*	−2.15 *−2.73*	−4.99 *−15.59*	−0.84 *−3.02*
		Methyl acetyl phosphonate (AcPMe) *with flexible residues*	5.77 *−15.12*	−2.73 *−3.20*	9.19 *−11.48*	−1.63 *−2.81*
		Dimethyl acetyl phosphonate (AcPMe_2_) *with flexible residues*	4.03 *−7.73*	−1.76 *−3.14*	−6.51 *−20.95*	−1.11 *−3.23*
		Ethyl acetyl phosphonate (AcPEt) *with flexible residues*	2.65 *−16.54*	−3.31 *−3.89*	3.19 *−20.32*	−2.38 *−3.96*
Branched-chain 2-oxo acid dehydrogenase (BCDH, EC 1.2.4.4)	Substrates	Pyruvate	−4.84	−1.85	−10.18	−2.71
		2-Oxobutyrate	−2.96	−0.75	−12.31	−2.71
		2-Oxoisovalerate	−8.57	−2.23	−13.89	−2.45
		2-Oxoisocaproate	−8.29	−2.18	−18.44	−2.23
		(S)-3-Methyl-2-oxovalerate (R)-3-Methyl-2-oxovalerate (allo-isomer)	−5.97 −10.09	−1.84 −1.67	−10.16 −18.12	−2.03 −2.52
	Inhibitors	Isobutyryl phosphonate	−1.60	−0.77	7.32	−0.04
		Methyl isobutyryl phosphonate	−10.17	−2.27	−17.47	−3.17
		Dimethyl isobutyryl phosphonate	−10.77	−2.88	−20.04	−3.06
		Isovaleryl phosphonate	15.28	2.25	−17.44	−2.56
		Methyl isovaleryl phosphonate	−8.86	−2.12	−14.39	−2.98
		Dimethyl isovaleryl phosphonate	−8.93	−2.89	−25.37	−2.90
		(S)-2-Methylbutyryl phosphonate (MBP) (R)-2-Methylbutyryl phosphonate	−3.41 −4.25	−1.76 −1.53	−19.33 −0.63	−3.72 −0.01
		Methyl (S)-2-methylbutyryl phosphonate (MBPMe) Methyl (R)-2-methylbutyryl phosphonate	−7.03 −6.73	−2.05 −1.21	−17.7 −24.29	−1.47 −3.44
		Dimethyl (S)-2-methylbutyryl phosphonate (MBPMe_2_) Dimethyl (R)-2-methylbutyryl phosphonate	−6.77 −7.25	−2.82 −0.95	−17.65 −19.23	−3.01 −0.40
		Diethyl (S)-2-methylbutyryl phosphonate (MBPEt_2_) Diethyl (R)-2-methylbutyryl phosphonate	−8.33 −12.58	−2.29 −1.95	−23.21 −30.05	−3.73 −3.15
2-Oxoglutarate dehydrogenase (OGDH, EC 1.2.4.2)	Substrates	2-Oxoglutarate	−24.75	−4.67	−25.19	−4.65
		2-Oxoadipate	−28.96	−5.11	−29.52	−5.12
	Inhibitors	Succinyl phosphonate (SP)	−25.56	−5.40	−26.82	−5.09
		Phosphonomethyl succinyl phosphonate (PMSP)	−24.47	−4.69	−29.76	−5.05
		Phosphonodimethyl succinyl phosphonate (PDSP)	−12.22	−2.72	−26.06	−2.89
		Phosphonoethyl succinyl phosphonate (PESP)	−28.31	−5.65	−30.71	−5.30
		Carboxyethyl succinyl phosphonate (CESP)	−35.02	−6.34	−34.76	−5.57
		Diethyl succinyl phosphonate (DESP)	−28.99	−4.23	−35.22	−4.14
		Triethyl succinyl phosphonate (TESP)	−7.77	−1.62	−36.40	−1.98
		Glutaryl phosphonate (GP)	−24.30	−4.61	−29.98	−5.30
Transketolase (TKT, EC 2.2.2.1)	Substrate	D-xylulose-5-phosphate	−29.20	−6.90	−28.67	−6.46

**Table 3:** Structures of the BCDH ligands and their hydroxy-variants, known from the literature and used in the docking simulations in this study.

Type of Ligand	Actual Ligand	Ligand for Docking in Gnina
Substrates	2-Oxoisovalerate 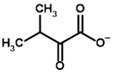	(S)-2-Hydroxyisovalerate 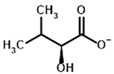
	2-Oxoisocaproate 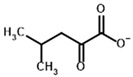	(S)-2-Hydroxyisocaproate 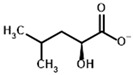
	3-Methyl-2-oxovalerate 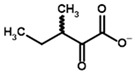	(2S)-2-Hydroxy-3-methylvalerate 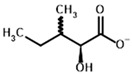
Inhibitors	Methyl 2-methylbutyryl phosphonate (MBPMe) 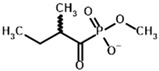	Methyl [(1R)-1-hydroxy-2-methylpropyl] phosphonate 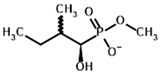
	Diethyl 2-methylbutyryl phosphonate (MBPEt_2_) 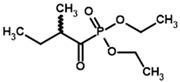	Diethyl [(1R)-1-hydroxy-2-methylpropyl] phosphonate 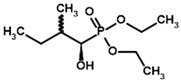

**Table 4:** Structures of the OGDH ligands and their hydroxy-variants, known from the literature and used in the docking simulations in this study.

Type of Ligand	Actual Ligand	Ligand for Docking in Gnina
Substrates	2-Oxoglutarate 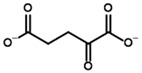	(S)-2-Hydroxyglutarate 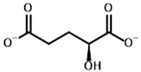
	2-Oxoadipate 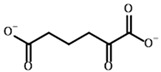	(S)-2-Hydroxyadipate 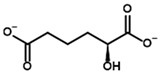
Inhibitors	Succinyl phosphonate (SP) 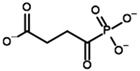	(R)-1-Hydroxy-3-carboxypropyl phosphonate 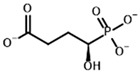
	Phosphonomethyl succinyl phosphonate (PMSP) 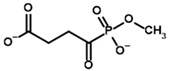	Methyl [(R)-1-Hydroxy-3-carboxypropyl] phosphonate 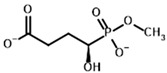
	Phosphonodimethyl succinyl phosphonate (PDSP) 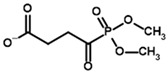	Dimethyl [(R)-1-Hydroxy-3-carboxypropyl] phosphonate 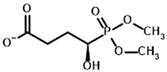
	Phosphonoethyl succinyl phosphonate (PESP) 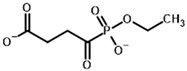	Ethyl [(R)-1-Hydroxy-3-carboxypropyl] phosphonate 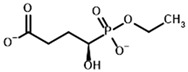
	Carboxyethyl succinyl phosphonate (CESP) 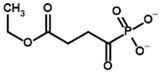	(R)-1-Hydroxy-3-ethylcarboxypropyl phosphonate 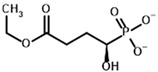
	Diethyl succinyl phosphonate (DESP) 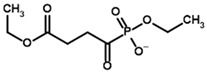	Ethyl (R)-1-Hydroxy-3-ethylcarboxypropyl phosphonate 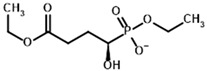
	Triethyl succinyl phosphonate (TESP) 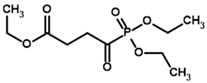	Diethyl (R)-1-Hydroxy-3-ethylcarboxypropyl phosphonate 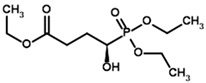
	Glutaryl phosphonate (GP) 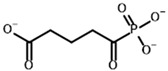	(R)-1-Hydroxy-4-carboxybutyryl phosphonate 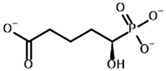

## Data Availability

Raw data are available from the authors upon request.

## Supplementary Materials

The following supporting information can be downloaded at: https://www.mdpi.com/article/10.3390/molecules30224427/s1, Figure S1: Comparison of optimal conformations upon covalent binding of pyruvate, 2-oxobutyrate and glyoxylate to ThDP-PDH complex using Gnina; Figure S2: Comparison of optimal conformations upon covalent binding of synthetic pyruvate analogs to ThDP-PDH complex using Gnina; Figure S3: Correlations of the binding affinities of synthetic pyruvate analogs towards PDH, estimated in various Gnina setups, with their inhibitory potential based on published K_i_ values; Figure S4: Comparison of optimal conformations upon covalent binding of BCDH substrates to ThDP-BCDH complex using Gnina; Figure S5: Comparison of optimal conformations upon covalent binding of synthetic 3-methyl-2-oxovalerate analogs to ThDP-BCDH complex using Gnina; Figure S6: Comparison of optimal conformations upon covalent binding of dicarboxylic 2-oxo acids to ThDP-OGDH complex using Gnina; Figure S7: Comparison of optimal conformations upon covalent binding of dicarboxylic 2-oxo acid phosphonate analogs to ThDP-OGDH complex using Gnina; Figure S8: Comparison of optimal conformations upon covalent binding of xylulose-5-phosphate to ThDP-TKT complex using various Gnina setups [167].

## Abbreviations

The following abbreviations are used in this manuscript:

OGDH	2-oxoglutarate dehydrogenase, or α-ketoglutarate dehydrogenase
TKT	Transketolase
PDH	pyruvate dehydrogenase
ThDP	thiamine diphosphate, or thiamine pyrophosphate, or cocarboxylase
BCDH	branched-chain 2-oxo acid dehydrogenase
HACL	2-hydroxyacyl-CoA-lyase
AHAS	acetohydroxyacid synthase
AcPH	acetyl phosphinate
AcMePH	acetyl (methyl) phosphinate
AcP	acetyl phosphonate
AcPMe	methyl acetyl phosphonate
AcPMe_2_	dimethyl acetyl phosphonate
AcPEt	ethyl acetyl phosphonate
SP	succinyl phosphonate
PMSP	phosphonomethyl succinyl phosphonate
PDSP	phosphonodimethyl succinyl phosphonate
PESP	phosphonoethyl succinyl phosphonate
CESP	carboxyethyl succinyl phosphonate
DESP	diethyl succinyl phosphonate
TESP	triethyl succinyl phosphonate
MBP	2-methylbutyryl phosphonate
MBPMe	methyl 2-methylbutyryl phosphonate
MBPMe_2_	dimethyl 2-methylbutyryl phosphonate
MBPEt_2_	diethyl 2-methylbutyryl phosphonate
GP	glutaryl phosphonate
PLP	pyridoxal-5′-phosphate
WSL	Windows Subsystem for Linux
