# Supplementary material for: Covalent Docking to the Active Sites of Thiamine Diphosphate-Dependent Enzymes
Source: Molecules. 2025 Nov 16;30(22):4427. doi: 10.3390/molecules30224427 (PMC12655727; doi:10.3390/molecules30224427)
Supplement: Supplementary file 1 [file molecules-30-04427-s001.zip › molecules-3933951-supplementary.pdf]

Supplementary Materials

Covalent Docking to the Active Site of Thiamine Diphosphate-Dependent Enzymes

Artem V. Artiukhov [1,2]*, Vasily A. Aleshin [1,2]

1 Department of Biokinetics, A. N. Belozersky Institute of Physicochemical Biology, Lomonosov Moscow State University, 119234 Moscow, Russia; whitelord32br@gmail.com, aleshinvasily@gmail.com

2 Department of Biochemistry, Sechenov University, 105043 Moscow, Russia

* Correspondence: whitelord32br@gmail.com; Tel.: +7-(495)-939-4484

Supplementary Figures

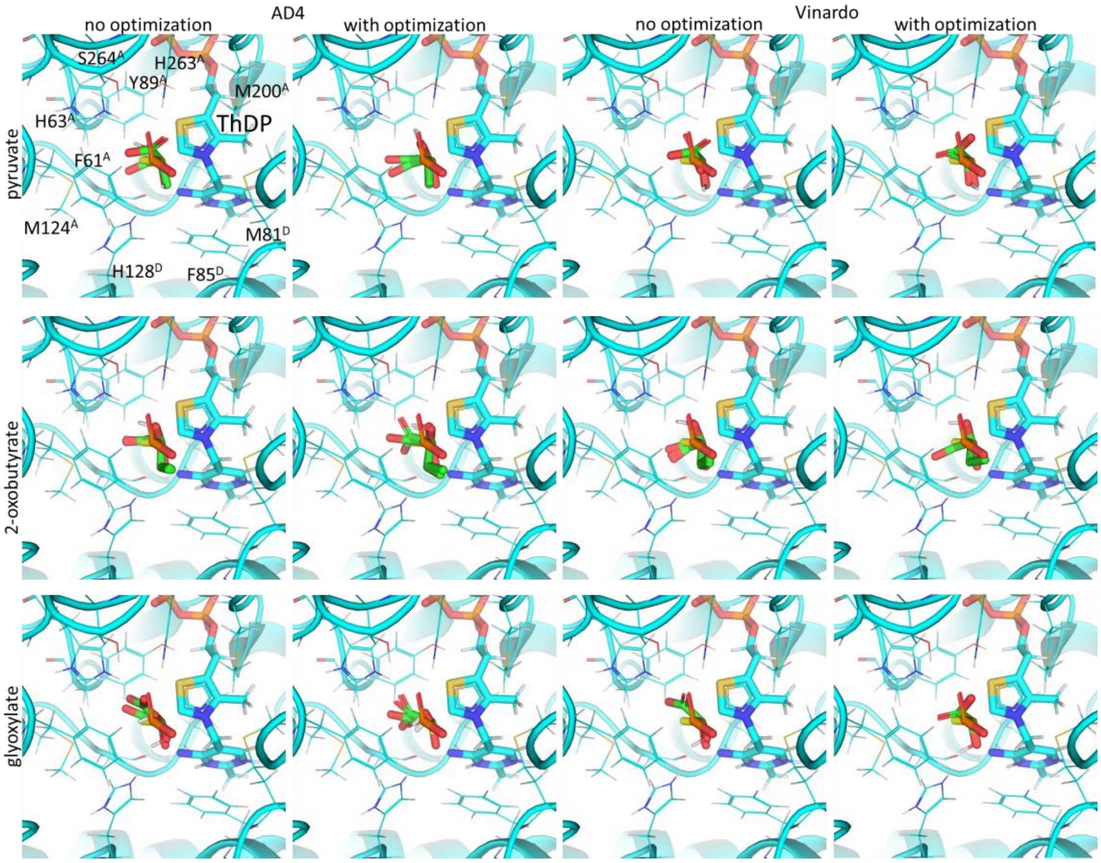

Supplementary Figure S1. Comparison of optimal conformations upon covalent binding of pyruvate, 2-oxobutyrate and glyoxylate to ThDP-PDH complex using Gnina. Docking poses were obtained using AD4 or Vinardo scoring functions with and without UFF ligand optimization. Only conformations with the lowest binding energy in each run are shown. Atom color scheme is the same as in Figure 2A, except carbon atoms of docked pyruvate, 2-oxobutyrate and glyoxylate are colored in green, and carbon atoms of AcPH initially present in PDH structure – in yellow. Amino acids residues within 6Å of the docked ligands are labelled; superscripted letters indicate protein chains.

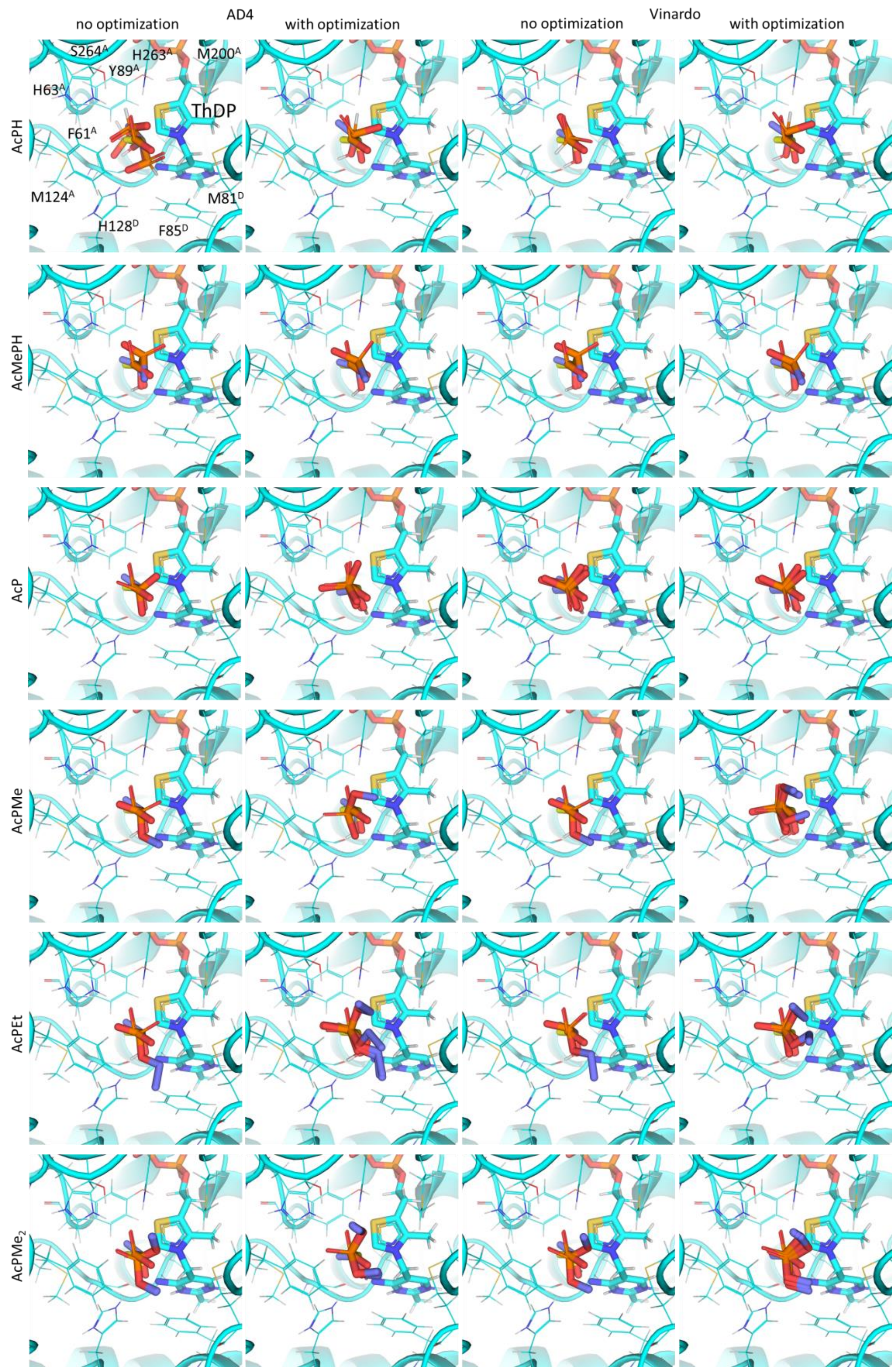

Supplementary Figure S2. Comparison of optimal conformations upon covalent binding of synthetic pyruvate analogs to ThDP-PDH complex using Gnina. Docking poses were obtained using AD4 or Vinardo scoring functions with and without UFF ligand optimization. Only conformations with the lowest binding energy in each run are shown. Atom color scheme and labels are the same as in Figure 4, except carbon atoms of docked pyruvate analogs are slate colored. Amino acids residues within 6Å of the docked ligands are labelled; superscripted letters indicate protein chains.

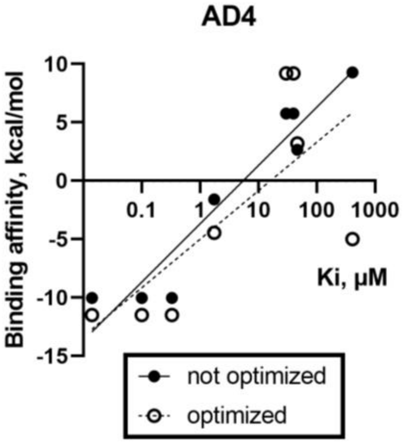

Semilog line (Ki is log, Energy is linear)

AD4	Yintercept	Slope	R^2
not optimized	–3.69±0.94	4.99±0.61	0.92
optimized	–5.01±2.52	4.15±1.63	0.52

Pearson correlations

	r	P-value	R^2
not optimized	0.96	<0.001	0.92
optimized	0.72	0.044	0.52

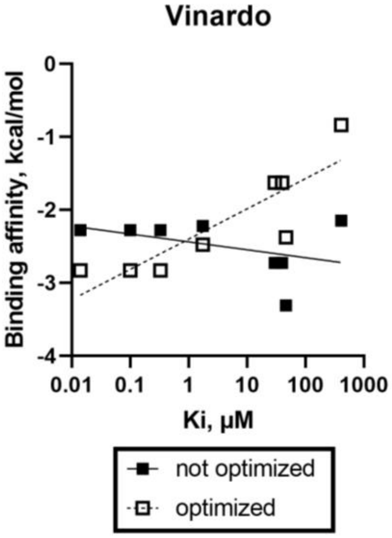

Semilog line (Ki is log, Energy is linear)

Vinardo	Yintercept	Slope	R^2
not optimized	–2.44±0.15	–0.11±0.10	0.18
optimized	–2.40±0.15	0.41±0.10	0.76

Pearson correlations

	r	P-value	R^2
not optimized	–0.42	0.298	0.18
optimized	0.87	0.005	0.76

Supplementary Figure S3. Correlations of the binding affinities of synthetic pyruvate analogs towards PDH, estimated in various Gnina setups, with their inhibitory potential based on published K_i values. Data on mammalian PDHs were from [1-4], data on related PDH from *B. stearothermophilus* were from [5], data on unrelated PDH from *E. coli* was excluded from the analysis. K_i values for same inhibitors, but from different papers were not merged. Semilog approximations and calculation of Pearson's correlation coefficients were performed in GraphPad Prism v.8.0 (GraphPad Software, Boston, USA).

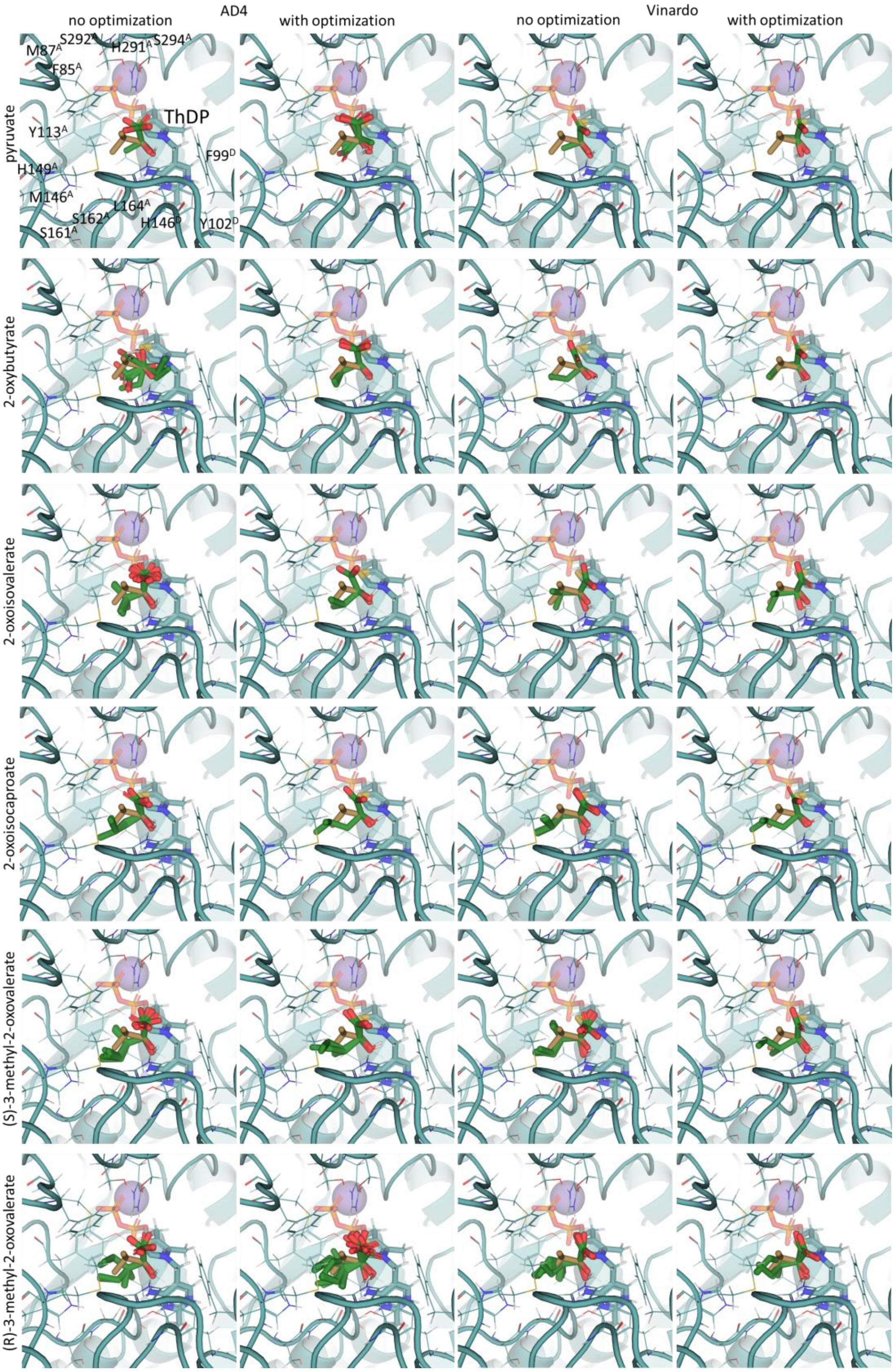

Supplementary Figure S4. Comparison of optimal conformations upon covalent binding of BCDH substrates to ThDP-BCDH complex using Gnina. Docking poses were obtained using AD4 or Vinardo scoring functions with and without UFF ligand optimization. Only conformations with the lowest binding energy in each run are shown. Carbon atoms of BCDH protein template from PDB ID: 2J9F (including ThDP and nearby amino acid residues) are colored in marine; carbon atoms of docked pyruvate, 2-oxobutyrate and branched-chain 2-oxo acids are in green, and carbon atoms of decarboxylated 2-oxoisovalerate initially present in BCDH structure are sand-colored; non-carbon atoms are colored according to standard color scheme. Amino acids residues within 6Å of the docked ligands are labelled; superscripted letters indicate protein chains.

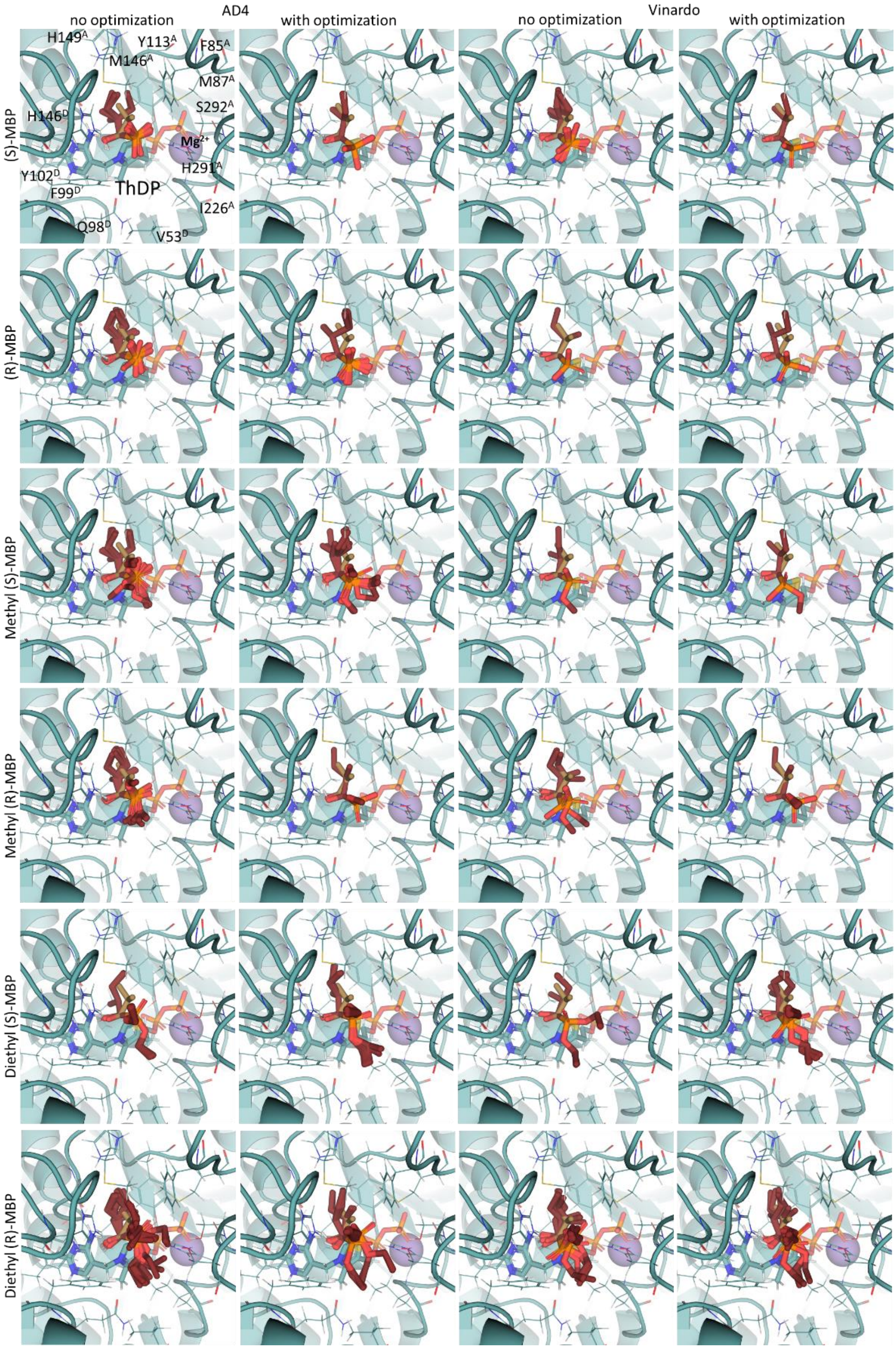

Supplementary Figure S5. Comparison of optimal conformations upon covalent binding of synthetic 3-methyl-2-oxovalerate analogs to ThDP-BCDH complex using Gnina. Docking poses were obtained using AD4 or Vinardo scoring functions with and without UFF ligand optimization. Only conformations with the lowest binding energy in each run are shown. Atom color scheme is the same as in Figure 6, except carbon atoms of docked (S)- and (R)-enantiomers of 2-methylbutyryl phosphonate (MBP) and their esters are firebrick-colored. Amino acids residues within 6Å of the docked ligands are labelled; superscripted letters indicate protein chains.

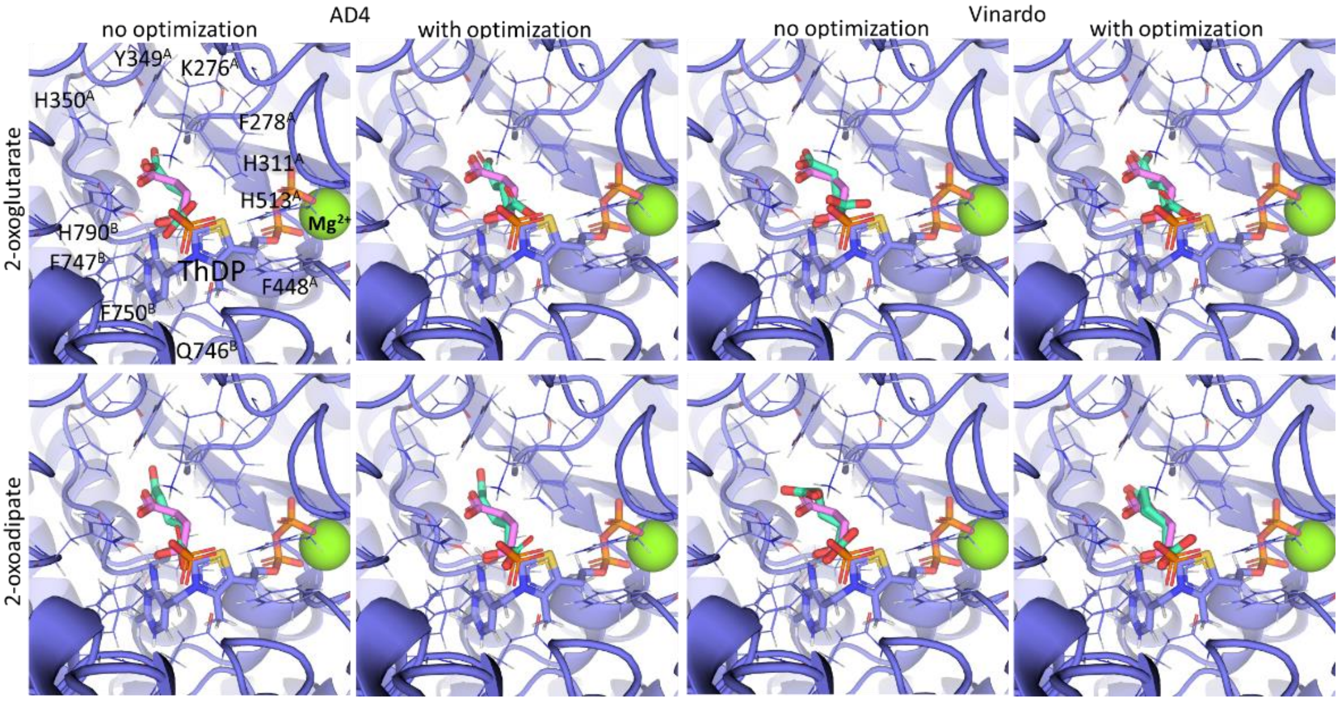

Supplementary Figure S6. Comparison of optimal conformations upon covalent binding of dicarboxylic 2-oxo acids to ThDP-OGDH complex using Gnina. Docking poses were obtained using AD4 or Vinardo scoring functions with and without UFF ligand optimization. Only conformations with the lowest binding energy in each run are shown. Carbon atoms of OGDH protein template homology modelled from PDB ID: 2YID and 6R29 (including ThDP and nearby amino acid residues) are indigo-colored; carbon atoms of docked 2-oxoglutarate and 2-oxoadipate are in aquamarine, and carbon atoms of SP initially present in OGDH structure are lilac-colored; non-carbon atoms are colored according to standard color scheme. Amino acids residues within 6Å of the docked ligands are labelled; superscripted letters indicate protein chains.

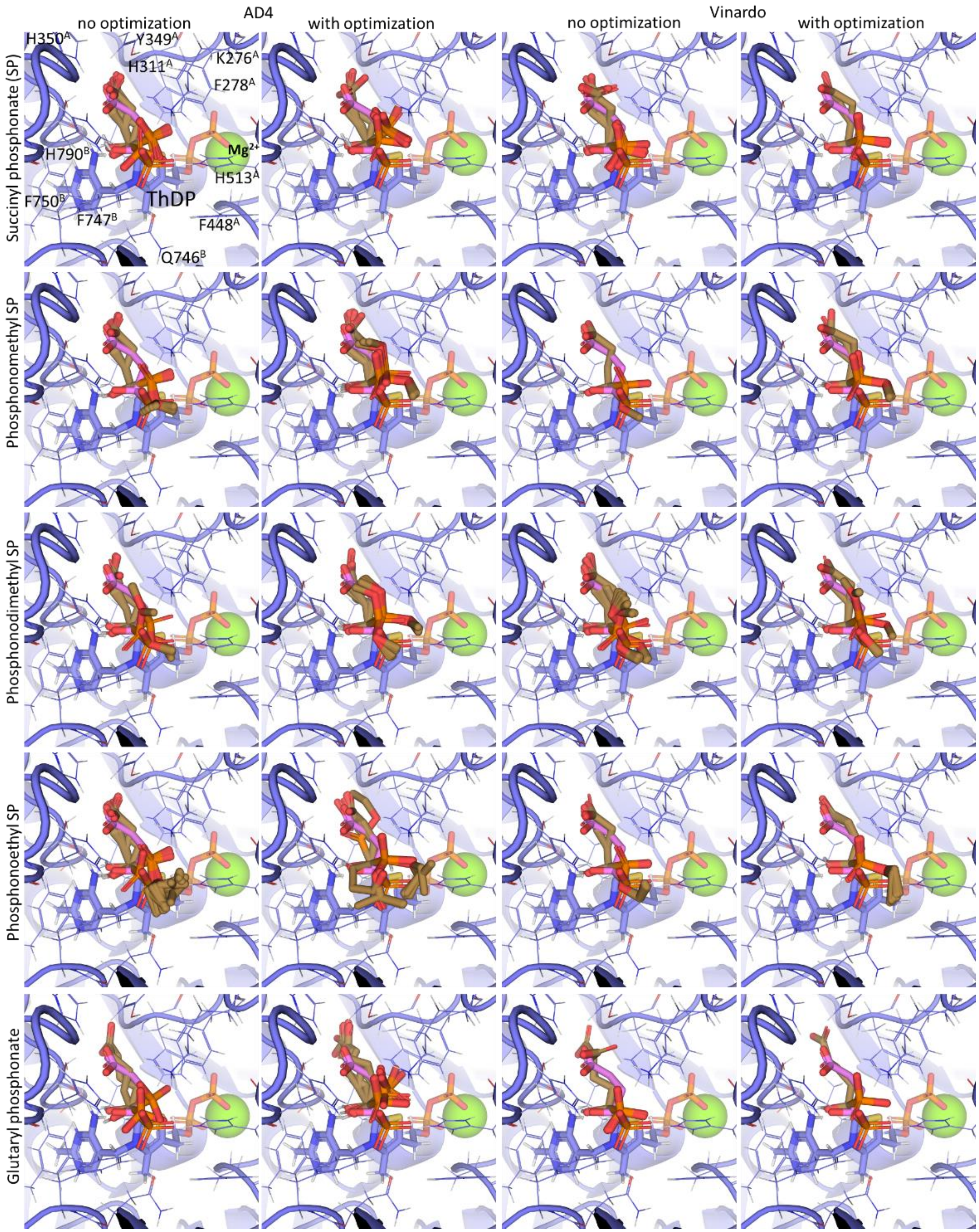

Supplementary Figure S7. Comparison of optimal conformations upon covalent binding of dicarboxylic 2-oxo acid phosphonate analogs to ThDP-OGDH complex using Gnina. Docking poses were obtained using AD4 or Vinardo scoring functions with and without UFF ligand optimization. Only conformations with the lowest binding energy in each run are shown. Atom color scheme is the same as in Figure 8, except carbon atoms of docked SP, its phosphonoesters, and GP are olive-colored. Amino acids residues within 6Å of the docked ligands are labelled; superscripted letters indicate protein chains.

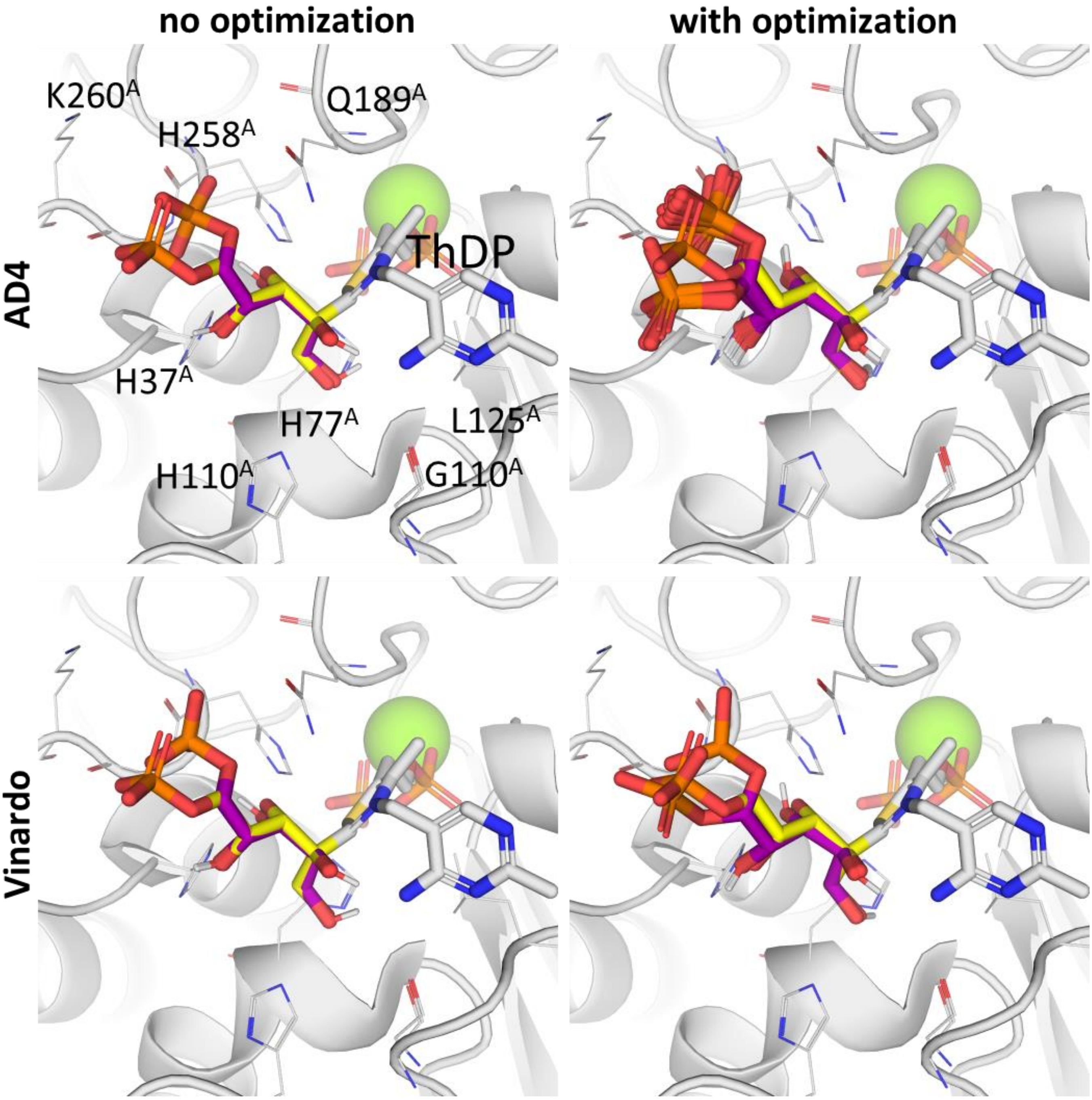

Suplementary Figure S8. Comparison of optimal conformations upon covalent binding of xylulose-5-phosphate to ThDP-TKT complex using various Gnina setups. Docking poses were obtained using AD4 or Vinardo scoring functions with and without UFF ligand optimization. Only conformations with the lowest binding energy in each run are shown. Carbon atoms of TKT protein template from PDB ID: 4KXW (including ThDP and nearby amino acid residues) are colored in grey; carbon atoms of docked xylulose-5-phosphate are in purple, and carbon atoms of decarboxylated 2-oxoisovalerate initially present in BCDH structure are lemon-colored; non-carbon atoms are colored according to standard color scheme. Amino acids residues within 6Å of the docked ligands are labelled; superscripted letters indicate protein chains.

References

1. Bunik, V.I.; Artiukhov, A.; Kazantsev, A.; Goncalves, R.; Daloso, D.; Oppermann, H.; Kulakovskaya, E.; Lukashev, N.; Fernie, A.; Brand, M., et al. Specific inhibition by synthetic analogs of pyruvate reveals that the pyruvate dehydrogenase reaction is essential for metabolism and viability of glioblastoma cells. *Oncotarget* **2015**, *6*, 40036-40052, doi:10.18632/oncotarget.5486.

2. Nemeria, N.S.; Korotchkina, L.G.; Chakraborty, S.; Patel, M.S.; Jordan, F. Acetylphosphinate is the most potent mechanism-based substrate-like inhibitor of both the human and Escherichia coli pyruvate dehydrogenase components of the pyruvate dehydrogenase complex. *Bioorganic chemistry* **2006**, *34*, 362-379, doi:10.1016/j.bioorg.2006.09.001.
3. Smith, J.M.; Vierling, R.J.; Meyers, C.F. Selective inhibition of E. coli 1-deoxy-D-xylulose-5-phosphate synthase by acetylphosphonates. *Medchemcomm* **2012**, *3*, 65-67, doi:10.1039/C1MD00233C.
4. Laber, B.; Amrhein, N. Metabolism of 1-aminoethylphosphinate generates acetylphosphinate, a potent inhibitor of pyruvate dehydrogenase. *The Biochemical journal* **1987**, *248*, 351-358.
5. Dixon, H.B.; Giddens, R.A.; Harrison, R.A.; Henderson, C.E.; Norris, W.E.; Parker, D.M.; Perham, R.N.; Slater, P.; Sparkes, M.J. A synthesis of acylphosphonic acids and of 1-aminoalkylphosphonic acids: the action of pyruvate dehydrogenase and lactate dehydrogenase on acetylphosphonic acid. *Journal of enzyme inhibition* **1991**, *5*, 111-117.

Disclaimer/Publisher's Note: The statements, opinions and data contained in all publications are solely those of the individual author(s) and contributor(s) and not of MDPI and/or the editor(s). MDPI and/or the editor(s) disclaim responsibility for any injury to people or property resulting from any ideas, methods, instructions or products referred to in the content.
